# Vaccination with Alpha-Gal Protects Against Mycobacterial Infection in the Zebrafish Model of Tuberculosis

**DOI:** 10.3390/vaccines8020195

**Published:** 2020-04-24

**Authors:** Iván Pacheco, Marinela Contreras, Margarita Villar, María Angeles Risalde, Pilar Alberdi, Alejandro Cabezas-Cruz, Christian Gortázar, José de la Fuente

**Affiliations:** 1 SaBio Instituto de Investigación en Recursos Cinegéticos IREC-CSIC-UCLM-JCCM, Ronda de Toledo s/n, 13005 Ciudad Real, Spain; ivan.pacheco@uclm.es (I.P.); marinela.contreras@uclm.es (M.C.); MargaritaM.Villar@uclm.es (M.V.); maria.alberdi@uclm.es (P.A.); christian.gortazar@uclm.es (C.G.); 2 Biochemistry Section, Faculty of Science and Chemical Technologies, and Regional Centre for Biomedical Research (CRIB), University of Castilla-La Mancha, 13071 Ciudad Real, Spain; 3 Departamento de Anatomía y Anatomía Patológica Comparadas, Facultad de Veterinaria, Universidad de Córdoba (UCO), Agrifood Excellence International Campus (ceiA3), 14071 Córdoba, Spain; mariaa.risalde@uclm.es; 4 UMR BIPAR, INRAE, ANSES, Ecole Nationale Vétérinaire d’Alfort, Université Paris-Est, 94700 Maisons-Alfort, France; cabezasalejandrocruz@gmail.com; 5 Department of Veterinary Pathobiology, Center for Veterinary Health Sciences, Oklahoma State University, Stillwater, OK 74078, USA

**Keywords:** alpha-Gal, vaccine, tuberculosis, vaccine, Mycobacterium, immunology

## Abstract

The alpha-Gal syndrome (AGS) is associated with tick bites that can induce in humans high levels of IgE antibodies against the carbohydrate Galα1-3Galβ1-(3)4GlcNAc-R (α-Gal) present in glycoproteins and glycolipids from tick saliva that mediate primarily delayed anaphylaxis to mammalian meat consumption. It has been proposed that humans evolved by losing the capacity to synthesize α-Gal to increase the protective immune response against pathogens with this modification on their surface. This evolutionary adaptation suggested the possibility of developing vaccines and other interventions to induce the anti-α-Gal IgM/IgG protective response against pathogen infection and multiplication. However, the protective effect of the anti-α-Gal immune response for the control of tuberculosis caused by *Mycobacterium* spp. has not been explored. To address the possibility of using vaccination with α-Gal for the control of tuberculosis, in this study, we used the zebrafish-*Mycobacterium marinum* model. The results showed that vaccination with α-Gal protected against mycobacteriosis in the zebrafish model of tuberculosis and provided evidence on the protective mechanisms in response to vaccination with α-Gal. These mechanisms included B-cell maturation, antibody-mediated opsonization of mycobacteria, Fc-receptor (FcR)-mediated phagocytosis, macrophage response, interference with the α-Gal antagonistic effect of the toll-like receptor 2 (TLR2)/nuclear factor kappa-light-chain-enhancer of activated B cells (NF-kB)-mediated immune response, and upregulation of pro-inflammatory cytokines. These results provided additional evidence supporting the role of the α-Gal-induced immune response in the control of infections caused by pathogens with this modification on their surface and the possibility of using this approach for the control of multiple infectious diseases.

## 1. Introduction

The alpha-Gal syndrome (AGS), which is now the focus of recent investigations, is associated with tick bites that can induce in humans high levels of IgE antibodies against the carbohydrate Galα1-3Galβ1-(3)4GlcNAc-R (α-Gal) present in glycoproteins and glycolipids from tick saliva that mediate delayed anaphylaxis to mammalian meat consumption and immediate anaphylaxis to tick bites, xenotransplantation, and certain drugs such as cetuximab [1,2,3,4,5,6,7,8,9,10,11,12]. Within the conflict and cooperation that drove the evolution of tick–host–pathogen interactions [13], humans evolved by losing the capacity to synthesize α-Gal to increase the protective immune response against pathogens with this modification on their surface while increasing the risk to develop the AGS [6]. This evolutionary adaptation suggested the possibility of developing vaccines and other interventions to induce the anti-α-Gal IgM/IgG protective response against pathogen infection to prevent or control major infectious diseases worldwide [14,15,16,17,18,19,20].

Recently, the C57BL/6 α1,3-Galactosyltransferase-knockout (α1,3-GalT-KO) mouse animal model was used to study the antibody response to the carbohydrate α-Gal and its potential for the control of infectious diseases such as malaria, leishmaniasis, Chagas disease, granulocytic anaplasmosis, and influenza caused by pathogens with this modification on their surface and/or by enhancing the protective immune response against these pathogens [15,16,17,18,19,20,21,22]. The results showed protection against pathogen infection in response to α-Gal-containing vaccine formulations [15,18,19,20,21,22]. However, the protective effect of the anti-α-Gal immune response for the control of tuberculosis caused by *Mycobacterium* spp. with α-Gal on their surface [17] and constituting one of the deadliest infectious diseases worldwide [23] has not been explored.

To address the possibility of using vaccination with α-Gal for the control of tuberculosis, in this study, we used the zebrafish *Danio rerio* (Hamilton, 1822) animal model. Zebrafish is a model organism for the study of immune mechanisms and new effective vaccines and control strategies against tuberculosis [24,25,26,27,28]. Additionally, zebrafish do not produce α-Gal and were recently shown to reproduce some features of the human immune response to this molecule as a model for the study of the AGS [29]. The results of this study showed that vaccination with α-Gal protects against mycobacterial infection in the zebrafish model of tuberculosis to further advance the possibility of developing a pan-vaccine for the simultaneous control of major infectious diseases worldwide [30]. Additionally, this vaccination strategy may be used for the control of fish mycobacteriosis or piscine tuberculosis affecting multiple freshwater and saltwater fish species and with human incidence worldwide [31].

## 2. Materials and Methods

### 2.1. Ethics Statement

Animal experiments were conducted in strict accordance with the recommendations of the European Guide for the Care and Use of Laboratory Animals. Animals were housed at and experiments were conducted at the experimental facility (IREC, Ciudad Real, Spain) with the approval and supervision of the Ethics Committee on Animal Experimentation of the University of Castilla La Mancha (PR-2018-06-13) and the Counseling of Agriculture, Environment, and Rural Development of Castilla La Mancha (ES130340000218).

### 2.2. Flow Cytometry Analysis of Mycobacterium marinum α-Gal Content

The *M. marinum* Aronson (ATCC 927) was cultured at 29 °C in 7H9 broth enriched with Middlebrook ADC (Becton Dickinson, Franklin Lakes, NJ, USA). The bacteria were washed twice in phosphate-buffered saline (PBS), centrifuges at 4000 g for 5 min, resuspended in PBS, fixed in 4% paraformaldehyde for 30 min at room temperature (RT), and washed once in PBS. The cells were incubated with 3% human serum albumin (HAS; Sigma-Aldrich, St. Louis, MO, USA) in PBS for 1 h at RT. Then, cells were incubated for 14 h at 4 °C with the α-Gal epitope (Galα1-3Galβ1-4GlcNAc-R) monoclonal antibody (M86, Enzo Life Sciences, Farmingdale, NY, USA) diluted 1:50 in 3% human serum albumin (HAS)/PBS. Fluorescein isothiocyanate (FITC)-goat anti-mouse IgM (Abcam, Cambridge, UK) labelled antibody (diluted 1/200 in 3% HSA/PBS) was used as a secondary antibody and incubated for 1 h at RT. Samples were analyzed on a FAC-Scalibur flow cytometer equipped with CellQuest Pro software (BD Bio-Sciences, Madrid, Spain). The viable cell population was gated according to forward-scatter (FSC-H) and side-scatter (SSC-H) parameters. Aliquots of fixed and stained samples were used for immunofluorescence assays after air-drying and mounting in ProLong Antifade reagent containing 4′,6-diamidino-2-phenylindole (DAPI) (Molecular Probes, Eugene, OR, USA). The sections were examined using a Zeiss LSM 800 laser scanning confocal microscope (Carl Zeiss, Oberkochen, Germany) with oil immersion objectives (63×).

### 2.3. Zebrafish

Wild-type adult (6–8 months old) AB female and male zebrafish were kindly provided by Juan Galcerán Sáez from the Instituto de Neurociencias (IN-CSIC-UMH, Sant Joan d’Alacant, Alicante, Spain). These zebrafish were certified by Biosait Europe S.L. (Barcelona, Spain; https://biosait.com) as free of major fish pathogens such as *Mycobacterium* spp., *Pseudoloma neurophilia*, *Pseudocapillaria tomentosa*, and zebrafish retroviruses. The zebrafish were maintained in a flow-through water system at 27 °C with a light/dark cycle of 14 h/10 h and fed twice daily with dry fish feed (Premium food tropical fish, DAPC, Valladolid, Spain).

### 2.4. Zebrafish Caccination With α-Gal and Challenge with M. marinum

#### 2.4.1. Experiment 1 (Figure 1A)

This experiment was designed to evaluate the effect of vaccination with bovine serum albumin (BSA) coated with α-Gal (BSA-α-Gal, thereafter named α-Gal; Dextra, Shinfield, UK) in combination with adjuvant Montanide ISA 71 VG (SEPPIC, Paris, France) and in comparison with BSA and adjuvant alone. For vaccine formulation, α-Gal was adjuvated as previously described [32] to a final concentration of 0.25 µg/µl in a vaccination dose volume of 20 µl. Fourteen fish were randomly allocated to vaccinated and control groups with a similar number of females and males. Fish were briefly anaesthetized by immersion in 0.02% tricaine methanesulfonate (MS-222) and vaccinated at weeks 0 (prime delivery) and 3 (boost delivery). Fish were vaccinated by intraperitoneal (IP) injection using a 30G insulin syringe with 50 µl of antigen composition. The *M. marinum* Aronson (ATCC 927) was cultured at 29 °C in 7H9 broth enriched with Middlebrook ADC (Becton Dickinson) and prepared for infection as previously described [27,33]. To verify the bacterial dose, *M. marinum* samples were diluted and plated on 7H10 agar enriched with Middelbrook OADC (Becton Dickinson) for counting bacterial colonies. Before challenge, fish were anaesthetized as described above and IP injected at week 5 with an infection dose equivalent to 50 ± 8 cfu of *M. marinum*, causing a chronic tuberculosis-like disease in zebrafish [27]. At week 8, fish were euthanized with immersion in 0.04% MS-222 and processed for analysis of antibody levels by ELISA, mycobacteria levels by qPCR, and expression of selected immune response gene markers by qRT-PCR. The zebrafish had weights of 333.0 ± 133.6 and 370.7 ± 91.2 mg at prime immunization and 344.2 ± 122.2 and 469.1 ± 106.0 mg at euthanasia for vaccinated and control groups, respectively.

#### 2.4.2. Experiment 2 (Figure 1B)

**Figure 1:**
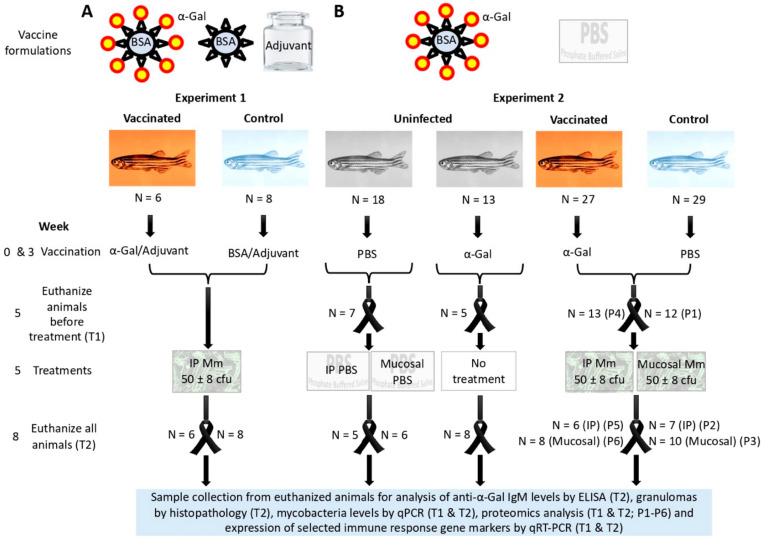
Experimental design: Two experiments were conducted to characterize the protective efficacy and mechanisms of vaccination with α-Gal in zebrafish infected with *M. marinum* (Mm). (**A**) In experiment 1, fish were parenterally (IP) vaccinated with α-Gal or bovine serum albumin (BSA) control with adjuvant and IP infected with Mm. Samples were collected after vaccination and treatment with Mm or PBS at the end of the experiment (T2). (**B**) Experiment 2 was conducted with IP vaccination with α-Gal without adjuvant in comparison with PBS-treated controls in fish infected with IP and mucosal Mm or left untreated. In experiment 2, samples were collected after vaccination and before treatment (T1) and at the end of the experiment (T2). Samples collected from euthanized fish were used for analysis of anti-α-Gal IgM levels by ELISA (T2), granulomas by histopathology (T2), mycobacteria levels by qPCR (T1 and T2), proteomics analysis (T1 and T2; P1–P6), and expression of selected immune response gene markers by qRT-PCR (T1 and T2).

In this experiment, the α-Gal antigen was formulated without adjuvant and used in comparison with PBS for vaccination of zebrafish treated with IP injection and mucosal exposure to PBS or *M. marinum* or left untreated. For vaccine formulation, α-Gal was diluted in PBS to a final concentration of 0.25 µg/µl in a vaccination dose volume of 20 µl. Controls were injected with a similar volume of PBS. A total of 87 fish were randomly allocated to uninfected/PBS, uninfected/α-Gal, vaccinated, and control groups with a similar number of females and males. Animals were vaccinated at weeks 0 and 3 as described in experiment 1. Then, at week 5 and before treatment (T1), fish from each group were euthanized as described above. Remaining fish were subjected to the different treatments at week 5 and euthanized at week 8 as described in experiment 1. These treatments included IP injection of PBS or *M. marinum* and mucosal exposure to PBS or *M. marinum*. IP injection was conducted as described above in experiment 1. Mucosal treatment was conducted as described before by immersion for 30 min in 500 ml water containing 56 ± 6 cfu/ml of *M. marinum*, while control animals were immersed in water with the same dose of PBS [28]. At week 8, fish were euthanized as described above in experiment 1 and processed for analysis of antibody levels by ELISA, granulomas by histopathology, mycobacteria levels by qPCR, proteome characterization by Sequential Windowed data independent Acquisition of the Total High-resolution Mass Spectra (SWATH), and expression of selected immune response gene markers by qRT-PCR (Figure 1B). The zebrafish had weights at prime immunization and euthanasia (T2), respectively, of 330.0 ± 132.0 and 345.9 ± 124.8 mg (PBS vaccination and treatment), 274.5 ± 137.3 and 357.9 ± 170.8 mg (α-Gal vaccination and no treatment), 258.3 ± 97.6 and 386.0 ± 78.3 mg (α-Gal vaccination and IP infection), 242.4 ± 117.9 and 323.4 ± 110.1 mg (α-Gal vaccination and mucosal infection), 279.4 ± 107.3 and 366.7 ± 149.3 mg (PBS vaccination and IP infection), and 340.4 ± 115.3 and 497.6 ± 84.0 mg (PBS vaccination and mucosal infection).

### 2.5. Anti-α-Gal IgM Antibody Titers in Zebrafish

For ELISA, high absorption capacity polystyrene microtiter plates were coated with 100 ng of α-Gal per well in carbonate-bicarbonate buffer (Sigma-Aldrich). After an overnight incubation at 4 °C, coated plates were washed one time with 100 µl/well PBS/1% Triton X-100 (PBST) (Sigma-Aldrich), and then blocked with 100 µl/well of 1% HSA (Sigma-Aldrich) for 1 h at RT. A dilution curve with 1:10, 1:100, and 1:1000 fish serum peritoneal fluid samples was performed and then diluted (1:10, v/v) in blocking solution, and 100 µl/well was added into the wells of the antigen-coated plates and incubated for 1.5 h at 37 °C. Plates were washed three times with PBST and 100 µl/well of species-specific rabbit anti-zebrafish IgM antibodies diluted (1:1000, *v*/*v*) in blocking solution were added and incubated for 1 h at RT. Plates were washed three times with 300 µl/well of PBST. A goat anti-rabbit IgG-peroxidase conjugate (Sigma-Aldrich) was added diluted 1:3000 in blocking solution and incubated for 1 h at RT. After four washes with 100 µl/well of PBST, 100 µl/well of 3,3′,5,5′-Tetramethylbenzidine (TMB) (Promega, Madison, WI, USA) was added and incubated for 15 min at RT. Finally, the reaction was stopped with 50 µl/well of 2 N H_2_SO_4_ and the optical density (OD) was measured in a spectrophotometer at 450 nm. The OD at 450 nm was compared between different groups by Student’s *t*-test with unequal variance (*p* < 0.05; experiment 1, *n* = 6 for vaccinated fish and *n* = 8 for controls; experiment 2, *n* = 6 for fish vaccinated and IP *M. marinum*, *n* = 8 for fish vaccinated and mucosal *M. marinum*, *n* = 7 for controls and IP *M. marinum*, and *n* = 10 for controls and mucosal *M. marinum*).

### 2.6. Histopathology

The analysis was conducted in animals collected from experiment 2 (Figure 1B). Zebrafish were sectioned sagittally, and half of them were immediately fixed in 10% neutral buffered formalin for 24 h at 21 °C, dehydrated in a graded series of ethanol, immersed in xylol, and embedded in paraffin wax using an automatic processor. Sections were cut at 4 µm and stained with haematoxylin and eosin (HE) and Ziehl–Neelsen (ZN) staining following standard procedures [27,28]. The small size of the zebrafish and the sagittal sections to divide them in two portions for molecular and histopathological studies permitted a histological assessment only in central nervous system, branchial arches, muscle, skin, liver, intestine, and gonads. Tuberculous granulomas were evaluated and classified according to their components (a) in early granuloma composed of an epithelioid macrophages infiltrate positive to mycobacteria and without or early necrosis and (b) in mature granuloma well-organized and infiltrated of epithelioid macrophages with areas of partial and complete central necrosis and acid-fast bacilli and insulated from the surrounding tissue by a fibrotic and/or cellular cuff. The quantitative assessment of the granulomas consisted of identifying and counting the organs with granulomas per zebrafish. Pathological findings were graded by a numerical score based on the number of granulomas, the type of granulomas, and the number of regions and/or organs involved by granulomatous disease in each fish. Histopathology and ZN staining for mycobacteria were used for the quantification of granuloma lesion scores in the studied organs. The granuloma lesion scores were compared between groups by Student’s *t*-test with unequal variance and by a one-way ANOVA test (https://www.socscistatistics.com/tests/anova/default2.aspx) and the number of early tuberculosis-like granulomas with epithelioid macrophages infiltrates surrounding scattered mycobacteria, and well-organized granulomas with partial and complete necrosis were compared between α-Gal vaccinated and not vaccinated zebrafish by Student’s t-test with unequal variance (*p* = 0.05; *n* = 5 for fish PBS vaccinated and IP PBS, *n* = 6 for fish PBS vaccinated and mucosal PBS, *n* = 7 for fish α-Gal vaccinated and untreated, *n* = 5 for fish vaccinated and IP *M. marinum*, *n* = 8 for fish vaccinated and mucosal *M. marinum*, *n* = 7 for controls and IP *M. marinum*, and *n* = 9 for controls and mucosal *M. marinum*).

### 2.7. Extraction of Total DNA, RNA, and Proteins from Zebrafish

Total DNA, RNA, and proteins were isolated from the intestine of euthanized fish using the AllPrep DNA/RNA/Protein (Qiagen, Hilden, Germany).

### 2.8. Characterization of M. marinum DNA Levels by qPCR

The DNA levels of *M. marinum* were determined by qPCR using the KAPA SYBR FAST one-step universal kit (Sigma-Aldrich) in the Rotor-Gene Q (Qiagen, Inc. Valencia, CA, USA) thermocycler following manufacturer’s recommendations with specific primers and conditions for *M. marinum 16S ribosomal RNA* (*16S rRNA*; Genbank accession number: AF456240.1) (16SForward-F: 5′-ACTGAGATACGGCCCAGACT-3′, 16SReverse-R: 5′-TCACGAACAACGCGACAAAC-3′, annealing 56 °C, 30 sec). A dissociation curve was run at the end of the reactions to ensure that only one amplicon was formed and that the amplicon denatured consistently in the same temperature range for every sample [34]. The DNA Ct values were normalized against *D. rerio glyceraldehyde-3-phosphate dehydrogenase* (*gapdh*; NM_001115114.1) (GAPDHF: 5′-CGTGGTGCCAGTCAGAACAT-3′, GAPDHR: 5′-AGTCAGTGGACACAACCTGG-3′, annealing 56 °C, 30 sec) using the genNormddCT method [35]. *M. marinum* DNA-normalized Ct levels were compared between groups by Student’s *t*-test with unequal variance (*p* = 0.05; experiment 1, *n* = 6 for vaccinated fish, *n* = 8 for controls; experiment 2 at T2, *n* = 6 for fish vaccinated and IP *M. marinum*, *n* = 8 for fish vaccinated and mucosal *M. marinum*, *n* = 7 for controls and IP *M. marinum*, and *n* = 10 for controls and mucosal *M. marinum*; experiment 2 at T1 vs. T2, *n* = 7 for PBS vaccinated uninfected fish, *n* = 5 for α-Gal vaccinated uninfected fish, *n* = 13 for α-Gal vaccinated infected fish, *n* = 12 for PBS vaccinated infected fish and T2, *n* = 5 for fish PBS vaccinated and IP PBS, *n* = 6 for fish PBS vaccinated and mucosal PBS, *n* = 8 for fish α-Gal vaccinated and untreated, *n* = 6 for fish vaccinated and IP *M. marinum*, *n* = 8 for fish vaccinated and mucosal *M. marinum*, *n* = 7 for controls and IP *M. marinum*, and *n* = 10 for controls and mucosal *M. marinum*). A Spearman’s Rho correlation analysis (https://www.socscistatistics.com/tests/spearman/Default2.aspx) was performed between normalized *M. marinum* DNA levels and anti-α-Gal IgM antibody levels (experiment 1, *n* = 6 for vaccinated fish, *n* = 8 for controls, ρ = −0.565, two-tailed *p* = 0.03; experiment 2, *n* = 14 for α-Gal vaccinated fish and IP/mucosal infection at T2, ρ = −0.515, two-tailed *p* = 0.004, *n* = 17 for PBS controls and IP/mucosal infection at T2, *n* = 6 for α-Gal vaccinated fish and IP infection at T2, *n* = 7 for PBS controls and IP infection at T2, ρ = −0454, two-tailed *p* = 0.12, and *n* = 8 for α-Gal vaccinated fish and mucosal infection at T2, *n* = 10 for PBS controls and mucosal infection at T2, ρ = −0522, two-tailed *p* = 0.03).

### 2.9. Characterization of mRNA Levels of Selected Zebrafish Immune Response Genes by qRT-PCR

To characterize the expression of selected genes previously shown to be involved in vaccine protective mechanisms [36] and/or fish immune response to infection [37], a qRT-PCR was performed for the analysis of *D. rerio tumor necrosis factor-alfa* (*tnf alpha*; NM_212859.2), *chemokine receptor type 4a* (*cxcr4a*; NM_131882.3), *chemokine receptor 6a* (*ccr6a*; NM_001099991.1), *toll-like receptor 2* (*TLR2*; NM_212812.1), *toll-like receptor 4* (*TLR4*; NM_001328605), *interleukin 1-beta* (*IL-1β*; NM_212844.2), *akirin 1* (*akr1*; NM_001007186.2), *akirin 2* (*akr2*; NM_213294.2), and *complement component 3* (*C3*; NM_131243.1) genes in zebrafish in response to vaccination and mycobacterial infection. The qRT-PCR was performed using the KAPA SYBR FAST one-step universal kit (Sigma-Aldrich) in the Rotor-Gene Q (Qiagen) with specifics forward (F) and reverse (R) primers and conditions following manufacturer’s recommendations (Table 1). A dissociation curve was run at the end of the reactions to ensure that only one amplicon was formed and that the amplicon denatured consistently in the same temperature range for every sample [34]. The mRNA Ct values were normalized against *D. rerio gapdh* as described above for the qPCR using the genNormddCT method [35]. The mRNA-normalized Ct values were compared between groups by Student’s *t*-test with unequal variance (*p* = 0.05) and then represented as the ratio between normalized Ct values in fish α-Gal-vaccinated and PBS control IP infected with *M. marinum* at T2 (*n* = 6 for fish α-Gal vaccinated and *n* = 7 for controls), the ratio between normalized Ct values in fish α-Gal-vaccinated and PBS control with mucosal infection with *M. marinum* at T2 (*n* = 8 for fish α-Gal vaccinated and *n* = 10 for controls), and the ratio between normalized Ct values at T1 and T2 (T1, *n* = 7 for PBS vaccinated uninfected fish, *n* = 5 for α-Gal vaccinated uninfected fish, *n* = 13 for α-Gal vaccinated infected fish, *n* = 12 for PBS vaccinated infected fish; T2, *n* = 5 for fish PBS vaccinated and IP PBS, *n* = 6 for fish PBS vaccinated and mucosal PBS, *n* = 8 for fish α-Gal vaccinated and untreated, *n* = 6 for fish α-Gal vaccinated and IP *M. marinum*, *n* = 8 for fish α-Gal vaccinated and mucosal *M. marinum*, *n* = 7 for controls and IP *M. marinum*, and *n* = 10 for controls and mucosal *M. marinum*).

### 2.10. Characterization of Zebrafish Proteome in Response to Vaccination and Infection

Isolated proteins were resuspended in 10 mM PBS with 2% SDS and protein concentration was determined using the BCA Protein Assay (Thermo Scientific) using bovine serum albumin (BSA) as standard. Proteins (100 μg per sample) were methanol/chloroform precipitated; resuspended in 30 μl 50 mM Tris-HCl pH 7.5, 2% SDS, 50 mM dithiothreitol (DTT); and boiled for 5 min at 95 C. Proteins were trypsin digested using the filter aided sample preparation (FASP) protocol using the FASP Protein Digestion Kit (Expedeon, San Diego, CA, USA) following manufacturer recommendations. The resulting peptides were desalted onto OMIX Pipette tips C18 (Agilent Technologies), dried-down; and stored at −20 °C until mass spectrometry analysis (MS) by SWATH-MS.

#### 2.10.1. Proteome Analysis by SWATH-MS

The desalted protein digests were resuspended in 2% acetonitrile and 5% acetic acid in water and analyzed by reverse phase liquid chromatography mass spectrometry (RP-LC-MS/MS) using an ekspertTM nanoLC 415 system coupled on line with a 6600 TripleTOF^®^ mass spectrometer (AB SCIEX; Framingham, MA, USA) through Information-Dependent Acquisition (IDA) followed by SWATH. Four micrograms of each protein digest of the different control and vaccinated groups from experiment 2 (four animals per group) were used for the generation of the reference spectral ion library as part of SWATH-MS analysis. The peptides were concentrated using a 0.1 × 20 mm C18 RP precolumn (Thermo Scientific), and then separated using a 0.075 × 250 mm C18 RP column (New Objetive, Woburn, MA, USA) operating at 0.3 ml/min. Peptides were eluted using a 120-min gradient from 5%–30% solvent B in solvent A followed by 15-min gradient from 30%–60% solvent B in solvent A (Solvent A: 0.1% formic acid in water, solvent B: 0.1% formic acid in acetonitrile) and directly injected into the mass spectrometer for analysis. For IDA experiments, the mass spectrometer was set to scanning full spectra (350–1400 *m*/*z*) using 250 ms accumulation time per spectrum, followed by up to 50 MS/MS scans (100–1500 *m*/*z*). Candidate ions with a charge state between +2 and +5 and counts per second above a minimum threshold of 100 were isolated for fragmentation. One MS/MS spectrum was collected for 100 ms, before adding those precursor ions to the exclusion list for 15 sec (mass spectrometer operated by Analyst TF 1.7; ABSciex). Dynamic background subtraction was turned off. MS/MS analyses were recorded in high-sensitivity mode with rolling collision energy on and a collision energy spread of 5. For SWATH quantitative analysis, twenty-two independent samples (four biological replicates from each PBS-vaccinated and α-Gal-vaccinated fish without treatment and with mucosal and IP *M. marinum* in experiment 2; Figure 1B) (8 μg each) were subjected to the cyclic data independent acquisition (DIA) of mass spectra using the SWATH variable windows calculator (V 1.0, AB SCIEX) and the SWATH acquisition method editor (AB SCIEX), similar to previously established methods [38]. A set of 50 overlapping windows was constructed (containing 1 *m*/*z* for the window overlap), covering the precursor mass range of 400–1250 *m*/*z*. For these experiments, a 50 -ms survey scan (350–1400 *m*/*z*) was acquired at the beginning of each cycle and SWATH-MS/MS spectra were collected from 100–1500 *m*/*z* for 70 ms at high-sensitivity mode, resulting in a cycle time of 3.6 sec. Collision energy for each window was determined according to the calculation for a charge +2 ion-centered upon the window with a collision energy spread of 15.

#### 2.10.2. Library Generation/Protein Identification, Data Processing, and Relative Quantitation

To create a spectral library of all the detectable peptides in the samples, the IDA MS raw files were combined and subjected to database searches in unison using ProteinPilot software v. 5.0.1 (AB SCIEX) with the Paragon algorithm [39]. Spectra identification was performed by searching against the *Danio rerio* Uniprot database (62,016 entries in February 2020) with the following parameters: iodoacetamide cysteine alkylation, trypsin digestion, identification focus on biological modification, and thorough ID as search effort. The detected protein threshold was set at 0.05. An independent False Discovery Rate (FDR) analysis, using the target-decoy approach provided by Protein Pilot (AB SCIEX), was used to assess the quality of identifications. Positive identifications were considered when identified proteins reached a 1% global FDR. For SWATH processing, up to 10 peptides with seven transitions per protein were automatically selected by the SWATH Acquisition MicroApp 2.0 in the PeakView 2.2 software (AB SCIEX) with the following parameters: 15 ppm ion library tolerance, 5 min extracted-ion chromatogram (XIC) extraction window, 0.01 Da XIC width, and considering only peptides with at least 99% confidence and excluding those which were shared or contained modifications. However, to ensure reliable quantitation, only proteins which had 3 or more peptides available for quantitation were selected for XIC peak area extraction and exported for analysis in the MarkerView 1.3 software (AB SCIEX). Global normalization was performed according to the total area sums of all detected proteins in the samples. In order to identify proteins that were significantly differentially represented between samples, a Student’s *t*-test (*p* = 0.05) was used to perform two-sample comparisons between the averaged area sums of all the transitions derived for each protein across the four replicate runs for each sample under comparison. Gene ontology (GO) annotations were obtained using the Blast2GO software (http://www.blast2go.org). Raw proteomics data was deposited at the PeptideAtlas repository (http://www.peptideatlas.org/) with the dataset identifier PASS01545.

## 3. Results

### 3.1. Experimental Design and Rationale

The experimental design used in this study addressed the protective efficacy of vaccination with α-Gal for the control of mycobacterial infection in the zebrafish model of tuberculosis using adjuvated (experiment 1; Figure 1A) and not adjuvated (experiment 2; Figure 1B) vaccine formulations. The rationale for using zebrafish is supported by constituting a validated model for tuberculosis [24,25,26,27,28] and the role of this species as an animal model for the study of the AGS [29]. The main objectives of the study were to characterize the effect of vaccination with α-Gal on mycobacterial infection and the immune mechanisms putatively involved in vaccine efficacy. To address these objectives, vaccinated and control zebrafish were infected with α-Gal-positive *M. marinum* [17], treated with PBS via IP or mucosal routes, or left untreated. Fish samples collected before and after treatment with *M. marinum* or PBS were used for analysis of IgM antibody levels by ELISA, granulomas by histopathology, mycobacteria levels by qPCR, proteomics analysis and expression of selected immune response gene markers by qRT-PCR (Figure 1A,B).

### 3.2. Zebrafish Antibody Response to Vaccination with α-Gal Correlates with Reduction in Mycobacterial Infection

**Figure 2:**
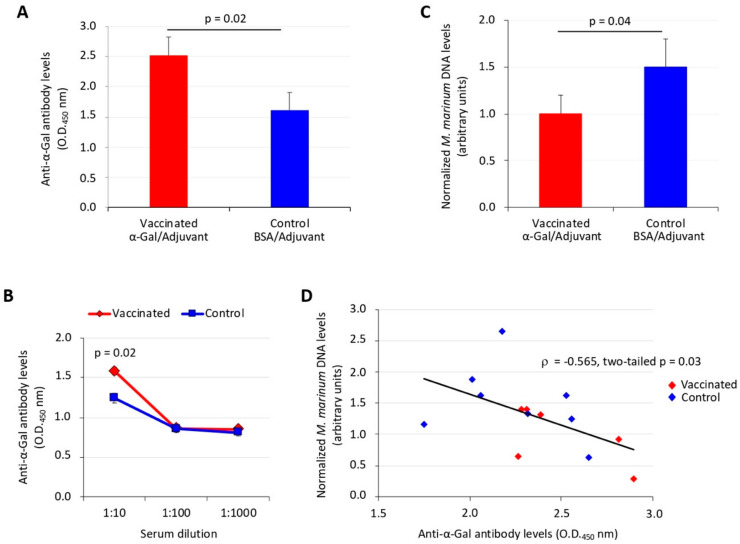
Experiment 1, effect of vaccination on the antibody response and mycobacterial infection levels in zebrafish. (**A**) The IgM antibody titers against α-Gal were determined by ELISA in fish vaccinated with adjuvated α-Gal or BSA alone as control and challenged with IP *M. marinum*. Antibody titers in vaccinated and control fish were represented as the average + SD of the OD_450nm_ (OD_antigen_ − OD_PBS control_), and the mean of the duplicate values were compared between vaccinated and control groups by Student’s *t*-test with unequal variance (*p* < 0.05). (**B**) ELISA dilution curve for IgM antibody titers against α-Gal in fish vaccinated with adjuvated α-Gal or BSA alone as control and challenged with *M. marinum*. Antibody titers were determined, represented, and analyzed as in (**A**) (*p* < 0.05). (**C**) *M. marinum* DNA levels were characterized by qPCR in vaccinated and control infected fish, normalized against *D. rerio gapdh*, represented as average + SD, and compared between groups by Student’s *t*-test with unequal variance (*p* < 0.05). (**D**) Spearman’s Rho correlation analysis between normalized *M. marinum* DNA levels and anti-α-Gal IgM antibody levels. Correlation rank coefficient (ρ) and *p*-value are shown. In all experiments, *n* = 6 for vaccinated fish and *n* = 8 for controls.

**Figure 3:**
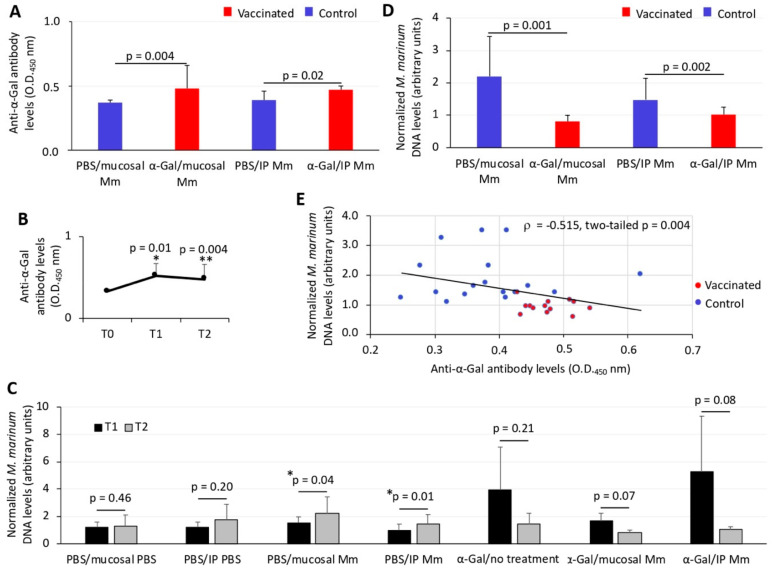
Experiment 2, effect of vaccination on the antibody response and mycobacterial infection levels in zebrafish. (**A**) The IgM antibody titers against α-Gal were determined by ELISA at T2 in fish vaccinated with α-Gal and PBS-treated controls infected with mucosal or IP *M. marinum* (Mm). Antibody titers in vaccinated and control fish were represented as the average + SD of the OD_450nm_ (OD_antigen_ − OD_PBS control_), and the mean of duplicate values were compared between vaccinated and control groups by Student’s *t*-test with unequal variance (*p* < 0.05; *n* = 6 for fish vaccinated and IP Mm, *n* = 8 for fish vaccinated and mucosal Mm, *n* = 7 for PBS controls and IP Mm, and *n* = 10 for PBS controls and mucosal Mm). (**B**) The IgM antibody titers against α-Gal were determined by ELISA at different time points in PBS-treated uninfected fish (T0 before vaccination, *n* = 18), vaccinated with α-Gal and uninfected fish (T1, *n* = 18) and vaccinated with α-Gal and untreated fish (T2, *n* = 8). Antibody titers were determined and represented as in (A) and compared by Student’s *t*-test with unequal variance (* *p* = 0.01, T1 vs. T0; ** *p* = 0.004, T2 vs. T0). (**C**) *M. marinum* DNA levels were characterized by qPCR at T1 and T2 in fish vaccinated with α-Gal and PBS-treated controls uninfected and infected with mucosal or IP Mm, normalized against *D. rerio gapdh*, represented as average + SD, and compared between T1 and T2 by Student’s *t*-test with unequal variance (* *p* < 0.05; T1: *n* = 7 for PBS vaccinated uninfected fish, *n* = 5 for α-Gal vaccinated uninfected fish, *n* = 13 for α-Gal vaccinated infected fish, *n* = 12 for PBS vaccinated infected fish; T2: *n* = 5 for fish PBS vaccinated and IP PBS, *n* = 6 for fish PBS vaccinated and mucosal PBS, *n* = 8 for fish α-Gal vaccinated and untreated, *n* = 6 for fish vaccinated and IP Mm, *n* = 8 for fish vaccinated and mucosal Mm, *n* = 7 for controls and IP Mm, and *n* = 10 for controls and mucosal Mm). (**D**) *M. marinum* DNA levels were characterized by qPCR at T2 in fish vaccinated with α-Gal and PBS-treated controls infected with mucosal or IP Mm, normalized against *D. rerio gapdh*, represented as average + SD, and compared between groups by Student’s t-test with unequal variance (*p* < 0.05; *n* = 6 for fish vaccinated and IP Mm, *n* = 8 for fish vaccinated and mucosal Mm, *n* = 7 for controls and IP Mm, and *n* = 10 for controls and mucosal Mm). (**E**) Spearman’s Rho correlation analysis between normalized *M. marinum* DNA levels and anti-α-Gal IgM antibody levels (experiment 2, *n* = 14 for α-Gal vaccinated fish and IP/mucosal infection at T2 and *n* = 17 for PBS controls and IP/mucosal infection at T2). Correlation rank coefficient (ρ) and *p*-value are shown.

In experiment 1 (Figure 1A), the anti-α-Gal IgM antibody levels significantly (*p* = 0.02) increased after vaccination with adjuvated α-Gal vaccine formulation when compared to controls (Figure 2A,B). The analysis of *M. marinum* DNA levels by qPCR showed a significant (*p* = 0.04) decrease in vaccinated when compared to control zebrafish (Figure 2C). A significant negative correlation (*p* = 0.03) was obtained between *M. marinum* DNA levels and anti-α-Gal IgM antibody titers (Figure 2D), thus providing evidence for the role of antibody response against α-Gal in the control of mycobacteriosis in vaccinated fish. In experiment 2 (Figure 1B), the results also showed a significant increase in the anti-α-Gal IgM antibody levels in both mucosal (*p* = 0.004) and IP (*p* = 0.02)-infected zebrafish when compared to controls (Figure 3A). The antibody response increased after vaccination (week 5, T1; *p* = 0.01) and remained higher than in untreated zebrafish (T0) until week 8 (T2; *p* = 0.004) (Figure 3B). The *M. marinum* DNA levels significantly increased after infection (T1 vs T2) only in unvaccinated PBS-treated and untreated control zebrafish for both mucosal (*p* = 0.04) and IP (*p* = 0.01) infection routes (Figure 3C). These results translated into a significant decrease in *M. marinum* DNA levels in α-Gal-vaccinated zebrafish when compared to controls infected via mucosal (*p* = 0.001) or IP (*p* = 0.002) routes (Figure 3D). As in experiment 1 but with a larger number of animals, a significant negative correlation (*p* = 0.004) was obtained between *M. marinum* DNA levels and anti-α-Gal IgM antibody titers (Figure 3E), thus providing additional support for the protective role of anti-α-Gal antibody response against mycobacterial infection in zebrafish.

### 3.3. The Tuberculous Granuloma Lesion Scores Decrease in Zebrafish Vaccinated with α-Gal and IP Infected with Mycobacteria

**Figure 4:**
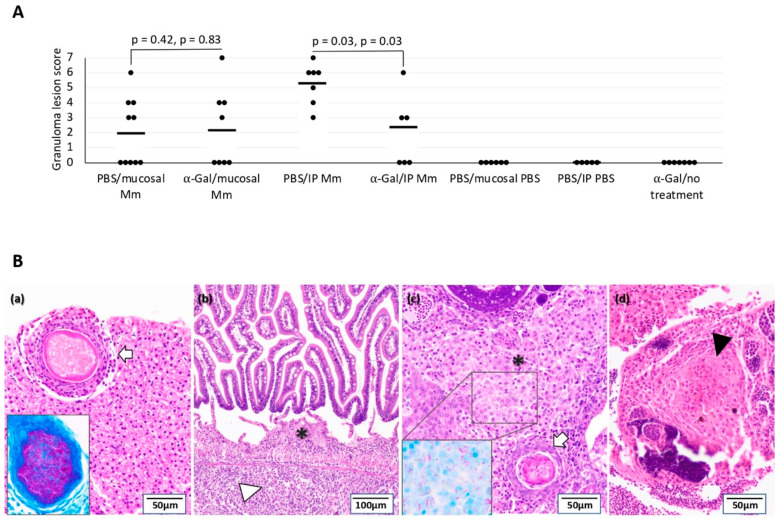
Effect of vaccination on tuberculous granulomas: The quantitative assessment of the granulomas consisted of identifying and counting the organs with granulomas per zebrafish. Pathological findings were graded by a numerical score based on the number of granulomas, the type of granulomas, and the number of regions and/or organs involved by granulomatous disease in each fish. (**A**) Histopathology and Ziehl–Neelsen (ZN) staining for mycobacteria were used for the quantification of granuloma lesion scores in the studied organs. The granuloma lesion scores were compared between groups by Student’s *t*-test with unequal variance and by a one-way ANOVA test (https://www.socscistatistics.com/tests/anova/default2.aspx) (*p* < 0.05; *n* = 5 for fish PBS vaccinated and IP PBS, *n* = 6 for fish PBS vaccinated and mucosal PBS, *n* = 7 for fish α-Gal vaccinated and untreated, *n* = 5 for fish vaccinated and IP *M. marinum*, *n* = 8 for fish vaccinated and mucosal *M. marinum*, *n* = 7 for controls and IP *M. marinum*, and *n* = 9 for controls and mucosal *M. marinum*). (**B**) Representative histopathology of zebrafish infected with *M. marinum*. (a) Liver tissue infected with *M. marinum* exhibiting tuberculosis-like mature granulomatous formation well-organized and surrounded by a capsule of connective tissue with complete central necrosis (white arrow). (b) Intestine from *M. marinum*-infected zebrafish presenting an early tuberculosis-like granuloma composed of epithelioid macrophages grouped loosely in lamina propia (asterisk *) and a diffuse epithelioid macrophages infiltrate in submucosa (white arrowhead). (c) Ovary tissue infected with *M. marinum* exhibiting an early tuberculosis-like granuloma composed of an epithelioid macrophage infiltrate (asterisk *) as well as a mature granuloma with well-defined border of macrophages and central necrosis (white arrow). (d) Testicle tissue from *M. marinum*-infected zebrafish showing a mature granuloma well-defined with early central necrosis (black arrowhead). A high acid-fast bacilli numbers were present in the central necrotic core of mature granulomas positive to ZN staining ((a), inset), while a lower bacterial number was observed residing within macrophages in early granulomas ((c), inset).

None of the animals in any group presented tuberculosis-like lesions at T0 and T1 of the experiment; neither did the animals from the *M. marinum* uninfected groups at T2. However, tuberculous-like granulomas were observed in 100% (IC_95%_: 61.2%–100%) of the animals from IP *M. marinum*-infected groups at T2 and in 50% (IC_95%_: 21.5%–78.4%) and 60% (IC_95%_: 31.2%–83.1%) of the zebrafish in mucosal *M. marinum*-infected groups vaccinated and not vaccinated with α-Gal, respectively. Fish vaccinated with α-Gal and infected IP with *M. marinum* had a significantly lower mean number of tuberculous granuloma lesion score (2 ± 3) when compared to unvaccinated animals (5 ± 1) (*p* = 0.03), while no significant differences between groups were observed in zebrafish infected by mucosal *M. marinum* (2 ± 2 vs. 2 ± 3; *p* > 0.40) (Figure 4A). However, all *M. marinum*-infected groups presented a similar granuloma distribution of affected tissues, with the liver and gonads the organs more affected followed by intestine (Figure 4B), whereas mycobacteria were not detected in the brain, branchial arches, muscle, and skin. ZN staining confirmed the presence of mycobacteria in the cytoplasm of macrophages within granulomas of all zebrafish with tuberculous-like lesions (insets in Figure 4B(a) and (c)).

Early tuberculosis-like granulomas with epithelioid macrophage infiltrates surrounding scattered mycobacteria were predominant in *M. marinum*-infected zebrafish in unvaccinated groups with respect to fish vaccinated with α-Gal, although no significant differences were observed in zebrafish infected by mucosal *M. marinum* (36.7% and 50.0% for vaccinated and unvaccinated groups, respectively; *p* = 0.69) and IP *M. marinum* (38.3% and 52.3% for vaccinated and unvaccinated groups, respectively; *p* = 0.46). Accordingly, a higher numbers of well-organized granulomas with partial and complete necrosis were present in not vaccinated and *M. marinum*-infected zebrafish but without significant differences within each group (25.8% and 50.0% for vaccinated and not vaccinated mucosal infected groups (*p* = 0.37) and 31.7% and 42.0% for vaccinated and not vaccinated IP infected groups (*p* = 0.53)). The acid-fast bacilli were found predominantly in necrotic areas and were in greater numbers in fully necrotic than in partially necrotic zones.

### 3.4. The α-Gal Content Varies among M. marinum Bacteria

**Figure 5:**
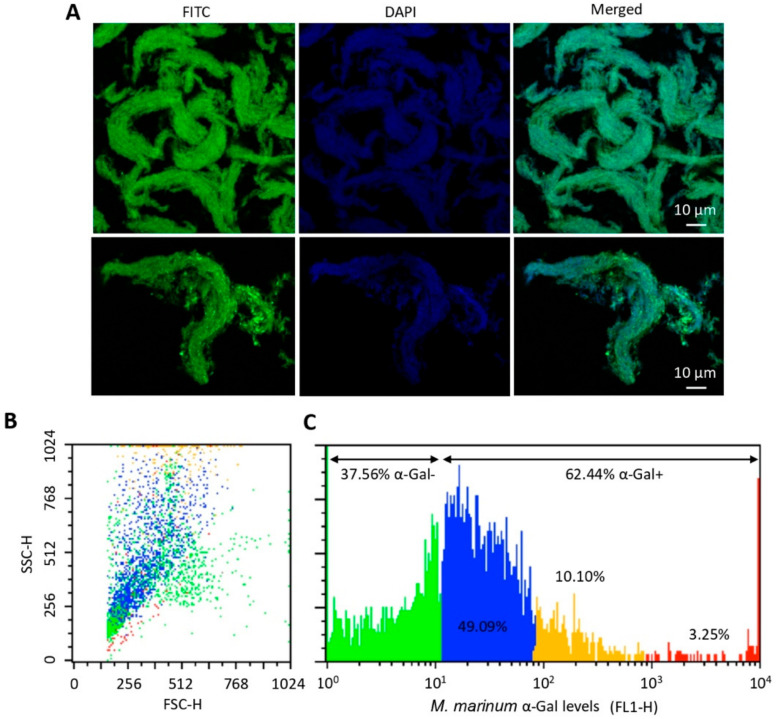
The α-Gal content in *M. marinum*: (**A**) Representative immunofluorescence images of *M. marinum* Aronson (ATCC 927) reference strain. (**B**) Density plot representing *M. marinum* that were gated by forward (FSC-H) and side (SSC-H) scatter. (**C**) Mycobacteria are represented in a histogram to evaluate the relative α-Gal levels (FL1-H). Cells were incubated with the α-Gal epitope monoclonal antibody M86. FITC-goat anti-mouse IgM-labelled antibody was used as a secondary antibody. Samples were analyzed on a FAC-Scalibur flow cytometer equipped with CellQuest Pro software. The viable cell population was gated according to forward-scatter (FSC-H) and side-scatter (SSC-H) parameters. Aliquots of fixed and stained samples were used for immunofluorescence assays after air-drying and mounting in ProLong Antifade reagent containing DAPI. The sections were examined using a Zeiss LSM 800 laser scanning confocal microscope with oil immersion objectives (63×; bars, 10 μm).

As previously shown [17], *M. marinum* has α-Gal on its surface (Figure 5A). However, the α-Gal content in *M. marinum* varied from negative or very low levels (37.56% of mycobacteria) to high levels (3.25% of mycobacteria) of α-Gal (Figure 5B,C). These results showed that not all mycobacteria have the same α-Gal content with possible functional implications.

### 3.5. The B-Cell Maturation and TLR2/NF-kB-Mediated Immune Responses Play a Role in Mycobacterial Infection and Protective Response to α-Gal in Zebrafish

**Figure 6:**
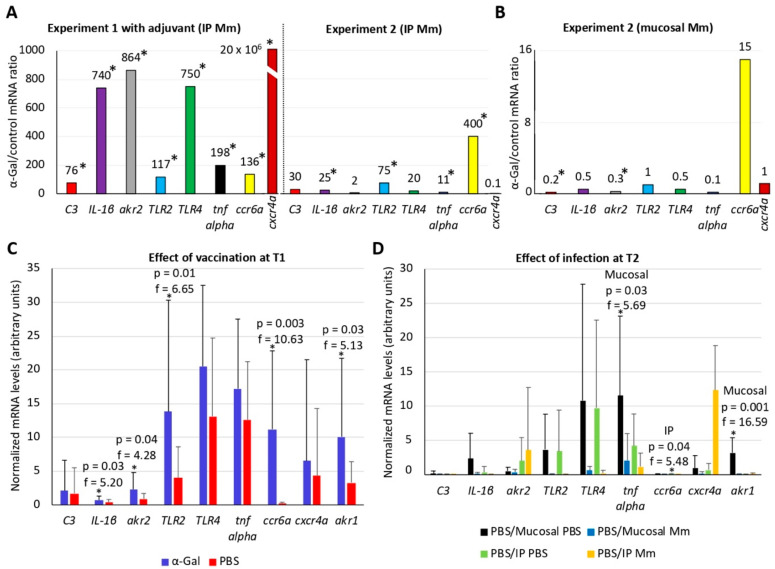
Effect of vaccination with α-Gal and mycobacterial infection on the expression of zebrafish immune response genes: The expression of selected immune response genes was characterized by qRT-PCR in zebrafish in response to vaccination with α-Gal or PBS and IP/mucosal infection with *M. marinum* (Mm). The mRNA levels were normalized against *D. rerio gapdh*, and normalized Ct values were compared between groups by Student’s *t*-test with unequal variance (* *p* < 0.05; Figure S1A,B) and then represented as (**A**) the ratio between normalized Ct values in fish α-Gal-vaccinated and PBS or BSA control IP infected with Mm at T2 (experiment 1, *n* = 6 for fish α-Gal vaccinated and *n* = 8 for controls; experiment 2, *n* = 6 for fish α-Gal vaccinated and *n* = 7 for controls) and (**B**) the ratio between normalized Ct values in fish α-Gal-vaccinated and PBS control with mucosal infection with Mm at T2 (*n* = 8 for fish α-Gal vaccinated and *n* = 10 for controls). (**C**) The normalized mRNA levels were compared between zebrafish vaccinated with α-Gal (*n* = 18) and PBS (*n* = 19) to evaluate the effect of vaccination at T1 by a one-way ANOVA test (https://www.socscistatistics.com/tests/anova/default2.aspx) (* *p* < 0.05). (**D**) The normalized mRNA levels were compared between zebrafish vaccinated with PBS and treated by mucosal or IP route with PBS or Mm to evaluate the effect of infection at T2 by a one-way ANOVA test (https://www.socscistatistics.com/tests/anova/default2.aspx) (* *p* < 0.05; *n* = 6 PBS/mucosal PBS, *n* = 10 PBS/mucosal Mm, *n* = 5 PBS/IP PBS, *n* = 7 PBS/IP Mm).

The expression of selected genes previously shown to be involved in vaccine protective mechanisms and/or fish immune response to infection was characterized by qRT-PCR in experiment 1 at T2 (after vaccination and treatment/IP infection) and in experiment 2 at T1 (after vaccination and before treatment/infection) and T2 (after vaccination and treatment/IP or mucosal infection) (Figure 6A–D and Figure S1A,B). The combined effect of vaccination and treatment/infection at T2 showed that the IP infection with *M. marinum* mostly upregulated the expression of selected genes with a higher effect on fish vaccinated with the adjuvant-containing α-Gal formulation (Figure 6A). In contrast, combined vaccination with mucosal *M. marinum* infection resulted in the downregulation or no effect of these genes (Figure 6B). In experiment 2, the effect of vaccination with α-Gal when compared to PBS-treated zebrafish at T1 resulted in significant (*p* < 0.05) upregulation of *IL-1β*, *akr2*, *TLR2*, *ccr6a*, and *akr1* (Figure 6C). In zebrafish infected with IP or mucosal *M. marinum*, the effect of infection when compared to PBS-treated controls at T2 showed a significant (*p* < 0.05) downregulation in response to infection for *tnf alpha* and *akr1* (mucosal infection) and *ccr6a* (IP infection) genes (Figure 6D).

To further evaluate the combined effect of vaccination and infection with *M. marinum* on zebrafish immune response genes, the mRNA levels of selected genes were compared at T1 and T2 in experiment 2 (Figures S2 and S3). The results showed that, as expected, the T1 = T2 mRNA ratio evidences no effect on immune response genes in fish vaccinated with PBS and treated with IP or mucosal PBS, thus validating the experimental design and analysis (Figures S2 and S3). The analysis of the different mRNA profiles showed that most genes had T1 = T2 and T1 > T2 profiles, thus suggesting no differences or a decrease in the mRNA levels after infection or no treatment in vaccinated fish (Figure S3).

After proteomics analysis, a total of 1777 proteins were identified in the zebrafish intestine (Table S1). Of them, differentially represented proteins after quantitation and comparative analysis by SWATH (*p* < 0.05) corresponded to 635 proteins (314 overrepresented and 321 underrepresented in P1 vs. P2, effect of IP infection at T2), 706 proteins (302 overrepresented and 404 underrepresented in P1 vs. P3, effect of mucosal infection at T2), 836 proteins (446 overrepresented and 390 underrepresented in P1 vs. P4, effect of vaccination at T1), 780 proteins (191 overrepresented and 589 underrepresented in P4 vs. P5, effect of vaccination and IP infection at T2), and 790 proteins (216 overrepresented and 574 underrepresented in P4 vs. P6, effect of vaccination and mucosal infection at T2) (Table S1). Although proteins encoded by most of the genes analyzed at the mRNA level were not identified in the proteomics analysis probably due to relatively low protein levels, these results showed an effect of the vaccination with α-Gal and/or mycobacterial infection in more than 60% of the identified proteins, suggesting a major impact on fish intestine proteome.

**Figure 7:**
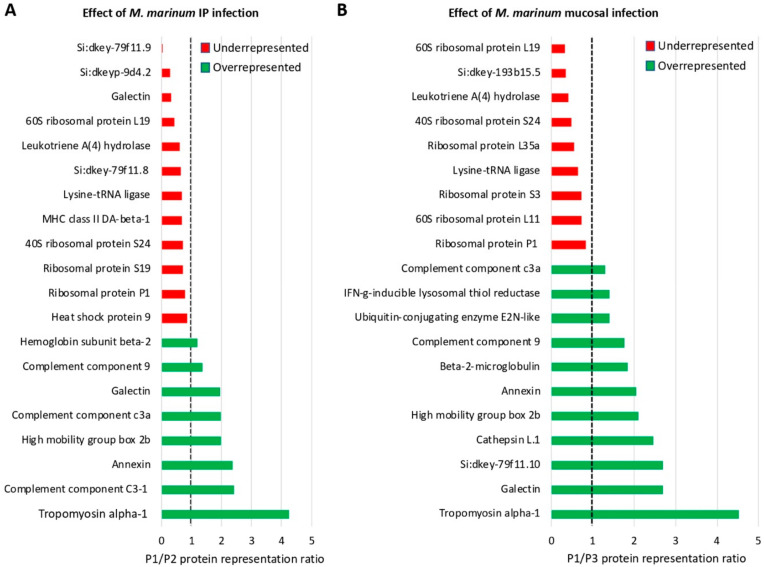
Proteomics analysis of immune system process proteins in response to *M. marinum* infection: Proteins annotated by Gene ontology (GO) using the Blast2GO software in the immune system process and significantly differentially represented between samples (Student’s *t*-test; *p* < 0.05, *n* = 4) were included. (**A**) Effect of *M. marinum* IP infection on protein representation (P1/P2 ratio) after comparison between PBS-vaccinated (P1 at T1; Figure 1B) and IP-infected (P2 at T2; Figure 1B) zebrafish. (**B**) Effect of *M. marinum* mucosal infection on protein representation (P1/P3 ratio) after comparison between PBS-vaccinated (P1 at T1; Figure 1B) and mucosal-infected (P3 at T2; Figure 1B) zebrafish.

**Figure 8:**
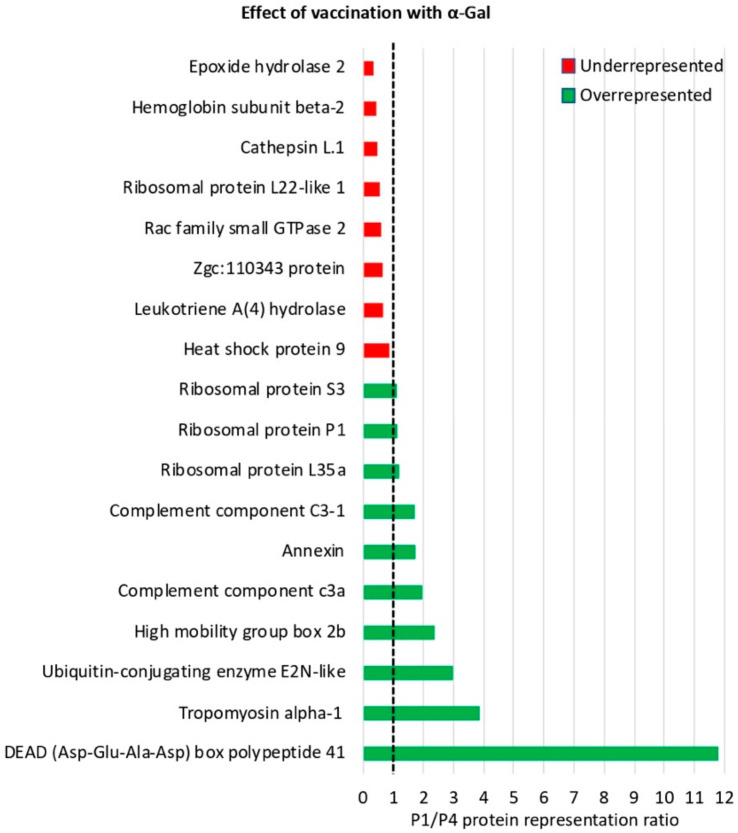
Proteomics analysis of immune system process proteins in response to vaccination with α-Gal: Proteins annotated by GO using the Blast2GO software in the immune system process and significantly differentially represented between samples (Student’s *t*-test; *p* < 0.05, *n* = 4) were included. Effect of vaccination with α-Gal on protein representation (P1/P4 ratio) after comparison between PBS-vaccinated (P1 at T1; Figure 1B) and α-Gal-vaccinated (P4 at T1; Figure 1B) zebrafish.

Proteomics analysis was focused on the immune system process proteins with special attention to those significantly represented (*p* < 0.05) in response to infection and vaccination (Figure 7A,B, Figure 8, Figures S4–S6). The effect of *M marinum* after IP (Figure 7A and Figure S4) and mucosal infection (Figure 7B and Figure S5) showed common overrepresented (e.g., ribosomal proteins, lysine-tRNA ligase, and leukotriene A4 hydrolase LTA4H) and underrepresented (e.g., complement components including C3, annexin, and tropomyosin alpha-1) proteins in response to infection (Figure 7A,B). Other proteins such as interferon (IFN)-g-inducible lysosomal thiol reductase, cathepsin L1, and heat shock protein 9 (HSP9) were differentially represented in response to either IP or mucosal infection (Figure 7A,B). The effect of vaccination with α-Gal evidenced the overrepresentation of proteins such as HSP9 and LTA4H, while complement components including C3, tropomyosin alpha-1, and annexin were underrepresented (Figure 8 and Figure S6). Other proteins such as ribosomal proteins were either under- or overrepresented in response to vaccination (Figure 8). Proteins such as DEAD (Asp-Glu-Ala-Asp) box polypeptide 41 (DDX41) and Rac family small GTPase 2 were underrepresented or overrepresented, respectively, in response to vaccination but did not change in response to infection (Figure 7A,B and Figure 8).

**Figure 9:**
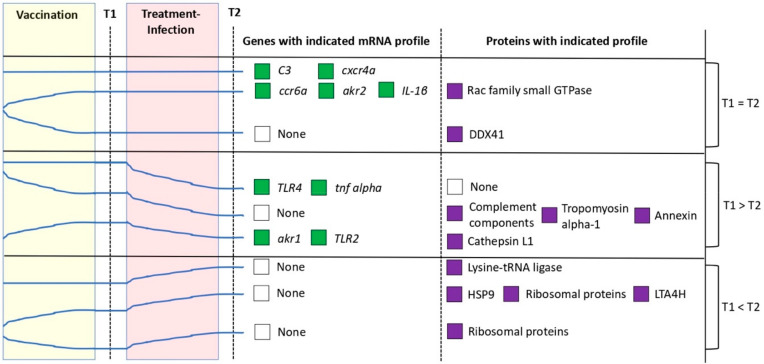
Summary mRNA and protein profiles of selected immune markers in response to vaccination and infection: The mRNA and protein profiles of selected immune response genes in response to vaccination at T1 and to treatment-infection at T2 are shown by tendency lines, which were associated to the corresponding genes or proteins. The results were compiled from qRT-PCR (Figure 6 and Figure S3) and proteomics (Figure 7A,B and Figure 8) analyses for experiment 2 at T1 and T2 (Figure 1B). Only statistically significant differences (*p* < 0.05) in mRNA and protein levels were considered. The results showed that the mRNA/protein levels of immune response markers involved in B-cell maturation (e.g., *ccr6a*), macrophage response (e.g., annexin), and TLR2/NF-kB-mediated response (e.g., *TLR2*, *akr1*, *akr2*, *tnf alpha*/*IL-1β*, DDX41, and Lysine-tRNA ligase) are downregulated by mycobacterial infection and upregulated in response to vaccination with α-Gal.

The analysis of the mRNA profiles showed that, while *C3* and *cxcr4a* mRNA levels did not change throughout the experiment, *ccr6a*, *akr2*, and *IL-1β* levels increased after vaccination with α-Gal and remained unchanged after infection (Figure 9). However, the mRNA levels for *TLR4*, *tnf alpha*, *akr1*, and *TLR2* remained unchanged or increased after vaccination but decreased after infection (Figure 9). The profiles of immune response proteins relevant for mycobacterial infection showed that LTA4H and cathepsin L1 levels increased in response to vaccination or vaccination and infection (Figure 9). However, the representation profile of DDX41 and lysine-tRNA ligase decreased and increased only in response to vaccination or infection, respectively (Figure 9). In contrast, the representation profile for complement components including C3, annexin, and tropomyosin alpha-1 decreased in response to vaccination and both IP and mucosal *M. marinum* infection (Figure 9). In summary, these results showed that the mRNA/protein levels of immune response markers involved in B-cell maturation (e.g., *ccr6a*) and TLR2/NF-kB-mediated response (e.g., *TLR2*, *akr1*, *akr2*, *tnf alpha*/*IL-1β*, LTA4H, and cathepsin L1) are downregulated/underrepresented, are overrepresented, or do no change in response to mycobacterial infection while upregulated/overrepresented in response to vaccination with α-Gal. The C3 protein levels showed underrepresentation in response to both vaccination and mycobacterial infection, a result that correlated with no effect on gene regulation (Figure 9).

## 4. Discussion

The possibility of using the antibody-mediated immune response against α-Gal for the control of infectious diseases caused by pathogens with this modification on their surface in hosts such as humans, birds, and fishes that do not have the capacity to synthesize α-Gal was initially suggested by results in the malaria mouse model [15]. Then, results in leishmaniasis and Chagas disease further supported this possibility [18,19,20], leading to proposing the possibility of development of a single-antigen pan-vaccine for the control of major infectious diseases worldwide [11,16,17,30]. Pathogens causing infectious diseases with high incidence worldwide and with α-Gal modifications include *Plasmodium*, *Mycobacterium*, *Leishmania*, *Trypanosoma*, *Anaplasma*, *Borrelia*, and *Aspergillus* species and viruses such as human immunodeficiency virus (HIV), measles virus, vaccinia virus, paramyxovirus, vesicular stomatitis virus, Sindbis virus, and retroviruses [6,9,14,15,17,18,19,20,21].

The mechanisms behind the possibility of using α-Gal for developing a single-antigen pan-vaccine for the control of infectious diseases caused by pathogens with this modification on their surface include pathogen opsonization by anti-α-Gal IgM/IgG-type antibodies and boosting the non-pathogen-specific protective immune mechanisms [10,14,15,16,17,18,19,20,21]. The immunization with α-Gal will increase the levels of the natural anti-α-Gal IgM/IgG-type antibodies produced in response to gut microbiota [15] and do not cause an increase in the IgE-type allergic response to tick saliva, which are involved in triggering the AGS [1,2,3,4,5,6,7,8,9,10,11,12,15,40]. Therefore, in principle, the α-Gal-based vaccines could be applied to all hosts that do not produce α-Gal, but the immune response could be affected by different factors including the ABO blood groups [17].

In this study, we provided additional support for this proposal by characterizing the protective effect of the anti-α-Gal immune response for the control of tuberculosis caused by *Mycobacterium* spp. using the zebrafish model. The zebrafish model of tuberculosis has been used in our laboratory for the characterization of the protective response elicited after vaccination with heat inactivated *M. bovis* (IV) [27,28]. In these experiments, we showed a reduction of mycobacteriosis in vaccinated fish and suggested that the innate immune response mediated by the C3 pathway activated through TLR-AKR2-IL-1β and other proinflammatory cytokines acted as the protective mechanism against infection. Herein, the results showed some similarities and differences in the immune mechanisms activated by vaccination with IV and α-Gal.

**Figure 10:**
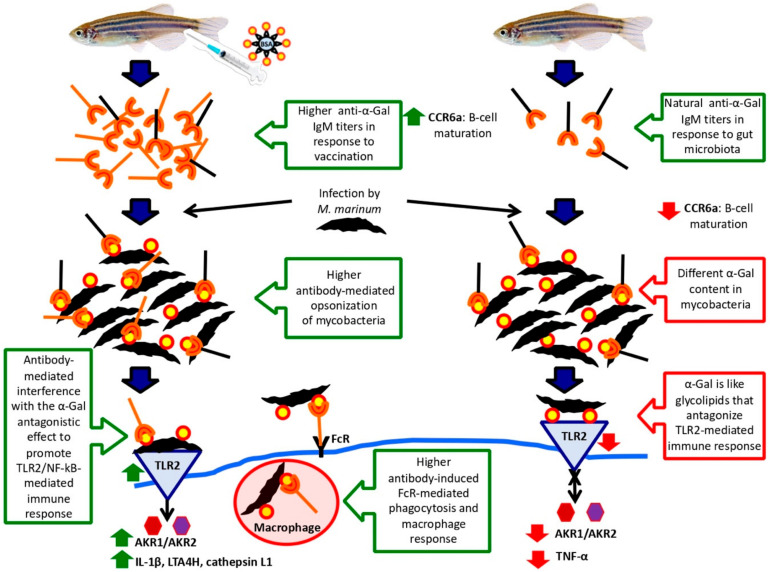
Immune mechanisms involved in mycobacterial infection and protective response to α-Gal in zebrafish: Zebrafish naturally produce anti-α-Gal IgM antibodies in response to gut microbiota with α-Gal modifications. However, vaccination with α-Gal results in higher anti-α-Gal IgM antibody levels. Additionally, while infection with *M. marinum* results in *ccr6a* downregulation, vaccination with α-Gal upregulates this gene to promote B-cell maturation and antibody production. Differences in α-Gal content between different mycobacteria may be an adaptive mechanism to prevent the anti-α-Gal IgM-mediated bacterial opsonization, which nevertheless will be more effective in vaccinated zebrafish. This mechanism will also interfere with the α-Gal antagonistic effect to TLR2-mediated immune response. In this way, while mycobacterial infection interferes with the TLR2/NF-kB pathway, the induction of the TLR2/NF-kB pathway in response to vaccination will promote the immunity and the FcR-mediated phagocytosis and macrophage response in vaccinated zebrafish to control mycobacteriosis.

The results suggested that *M. marinum* affects the zebrafish immunity by downregulating the expression of immune response genes with a stronger effect after mucosal infection that reproduces better the natural infection conditions. However, alternative innate immune mechanisms may be activated in response to mycobacterial infection [41]. Similar to vaccination with IV [27,28], the upregulation of proinflammatory cytokines through the TLR/NF-kB-AKR pathway was shown to be an α-Gal-induced putative protective mechanism to mycobacterial infection [42,43,44] (Figure 10). The use of adjuvant-containing α-Gal formulation showed higher levels of immune response genes in response to vaccination and infection when compared to α-Gal alone, thus suggesting that adjuvants may be considered to improve vaccine efficacy. However, the C3 pathway proposed to be involved in protective response to IV was not activated in α-Gal-vaccinated zebrafish. These results suggested that mycobacterial α-Gal may be like glycolipids that antagonize TLR2-mediated response (Figure 10). Cell envelope glycolipids and particularly sulfoglycolipids has been shown to inhibit NF-κB/AKR activation and subsequent cytokine production by acting as competitive antagonists of TLR2, thereby inhibiting the recognition of mycobacteria by this receptor [45].

The previously shown role of antibodies against mycobacterial surface-exposed antigens in the control of tuberculosis [46] was also supported by results of our study. Zebrafish are α-Gal negative and have natural anti-α-Gal antibodies in response to gut microbiota [29]. Mycobacteria contain α-Gal on their surface, and therefore, antibodies against this antigen can opsonize *M. marinum* and promote Fc-receptor (FcR)-mediated phagocytosis and macrophage response with a higher effect in vaccinated zebrafish with higher anti-α-Gal antibody levels (Figure 10). The expression of the gene coding for the CCR6a beta chemokine receptor, which has been implicated in B-lineage maturation and antigen-driven B-cell differentiation and humoral immunity [47], was downregulated in response to infection but upregulated in vaccinated zebrafish, thus promoting the production of anti-α-Gal antibodies (Figure 10). Furthermore, *tnf alpha* was downregulated by *M. marinum* infection but not after vaccination with α-Gal, thus supporting a role for this cytokine in augmenting cell-mediated immunity in vaccinated zebrafish [46] (Figure 10). Antibody-mediated macrophage activation increases TNF-α secretion, which plays a major role in macrophage recruitment to the infection site during the initial and long-term control of tuberculosis [48]. Remarkably, the results showed that not all mycobacteria have the same α-Gal content, which may constitute an adaptation to escape from the anti-α-Gal antibody protective response in infected hosts. Nevertheless, higher anti-α-Gal antibody levels in vaccinated fish may mediate the interference with the α-Gal antagonistic effect to promote TLR2-mediated immune response (Figure 10).

Despite the finding that fish vaccinated with α-Gal showed a decrease in *M. marinum* infection levels, only animals infected with IP *M. marinum* had a significantly lower number of tuberculous granuloma lesions when compared to unvaccinated animals, and all *M. marinum*-infected groups showed a similar granuloma distribution of affected tissues. The chemokine receptor CXCR4 promotes granuloma formation and induction of angiogenesis by *M. marinum* [49]. Furthermore, *cxcr4* mRNA levels increase in patients with tuberculosis whereas amelioration of disease reduces receptor expression in vivo [50]. The C3 cleavage fragments modulate CXCR4-mediated response [51,52]; TNF-α, which stimulates the production of C3 [53], is also important in granuloma formation; and its neutralization results in the loss of granuloma structure [47]. In zebrafish, *cxcr4* mRNA levels did not change in response to vaccination with α-Gal and infection with *M. marinum*, and *tnf alpha* was downregulated in response to infection (Figure 6A–D and Figure 7). These results supported that vaccination with α-Gal decreases mycobacterial infection by mechanisms not mediated by the C3 pathway that has been proposed to be involved in protective response to vaccination with IV [27,28,36,54].

## 5. Conclusions

Vaccination with α-Gal protected against mycobacteriosis in the zebrafish model of tuberculosis. The results provided evidence that the protective mechanisms in response to vaccination with α-Gal include B-cell maturation, antibody-mediated opsonization of mycobacteria, FcR-mediated phagocytosis, macrophage response, interference with the α-Gal antagonistic effect of the TLR2/NF-kB-mediated immune response, and upregulation of pro-inflammatory cytokines. These mechanisms result in the decrease of mycobacteria levels through the activation of the humoral and cellular immune responses. These results provided additional evidence supporting the role of the α-Gal-induced immune response in the control of infections caused by pathogens with this modification on their surface and the possibility of using this approach for the control of multiple infectious diseases. The fact that vaccination with IV and α-Gal activate different immune protective mechanisms suggested that it may be possible to combine these antigens in future experiments to increase vaccine efficacy against mycobacterial infection.

## Figures and Tables

**Table 1:** Oligonucleotide primer sequences and annealing conditions.

Gene	Oligonucleotide Primers	Annealing Conditions
*tnf alpha*	F: 5′-GCTTATGAGCCATGCAGTGA-3′	56 °C, 30 sec
	R: 5′-TGCCCAGTCTGTCTCCTTCT-3′	
*ccr6a*	F: 5′-AGCTTCTGCGTGGCATCTAT-3′	56 °C, 30 sec
	R: 5′-CAGACGGCTGCACAAACTAA-3′	
*TLR4*	F: 5′-TCACCTGGACAGCAAGAATG-3′	56 °C, 30 sec
	R: 5′-CGATTGACTTCCCTGCTTGA-3′	
*IL-1β*	F: 5′-GCATGTCCACATATGCGTCG-3′	58 °C, 30 sec
	R: 5′-GCTGGTCGTATCCGTTTGGA-3′	
*akr1*	F: 5′-AGTTTGAGGCCCTTCTCAGC-3′	58 °C, 30 sec
	R: 5′-AAGTGCCTTCATGTCTGGGG-3′	
*TLR2*	F: 5′-TGAATGGGTCGAGGAGATTC-3′	56 °C, 30 sec
	R: 5′-CACAAAGTGCTCCGACAGAA-3′	
*cxcr4a*	F: 5′-TGTACAGCAGCGTCCTCATC-3′	58 °C, 30 sec
	R: 5′-ACCCAGGTGACAAACGAGTC-3′	
*C3*	F: 5′-ACGCTCTCTGGATTGAAACA-3′	56 °C, 30 sec
	R: 5′-TGCCTTCTTGCATGGCAATC-3′	
*akr2*	F: 5′-ACTATGGACTTCGATCCGCT-3′	56 °C, 30 sec
	R: 5′-GCTCTGTGGTGAGTGCTGAA-3′	

## Supplementary Materials

The following are available online at https://www.mdpi.com/2076-393X/8/2/195/s1, Figure S1: Effect of zebrafish vaccination with α-Gal and mycobacterial infection on the expression of immune response genes in experiments 1 (Figure S1A) and 2 (Figure S1B), Figure S2: Effect of different treatments/vaccination and mycobacterial infection on the T1 to T2 mRNA ratio of immune response genes, Figure S3: mRNA profile of immune response genes in response to different treatments/vaccination and mycobacterial infection, Figure S4: Differentially represented proteins in response to mycobacterial IP infection at T2, Figure S5: Differentially represented proteins in response to mycobacterial mucosal infection at T2, Figure S6: Differentially represented proteins in response to vaccination with α-Gal at T1, Table S1: Proteomics results.
