# Supplementary material for: Vaccination with Alpha-Gal Protects Against Mycobacterial Infection in the Zebrafish Model of Tuberculosis
Source: Vaccines (Basel). 2020 Apr 24;8(2):195. doi: 10.3390/vaccines8020195 (PMC7348772; doi:10.3390/vaccines8020195)
Supplement: Supplementary file 1 [file vaccines-08-00195-s001.zip › Supplementary Materials.pdf]

Vaccination with α-Gal protects against mycobacterial infection in the zebrafish model of tuberculosis

Iván Pacheco, Marinela Contreras, Margarita Villar, María Angeles Risalde, Pilar Alberdi, Alejandro Cabezas-Cruz, Christian Gortázar, José de la Fuente

Supplementary Materials:

Figures S1A, S1B, S2, S3, S4, S5 and S6

A

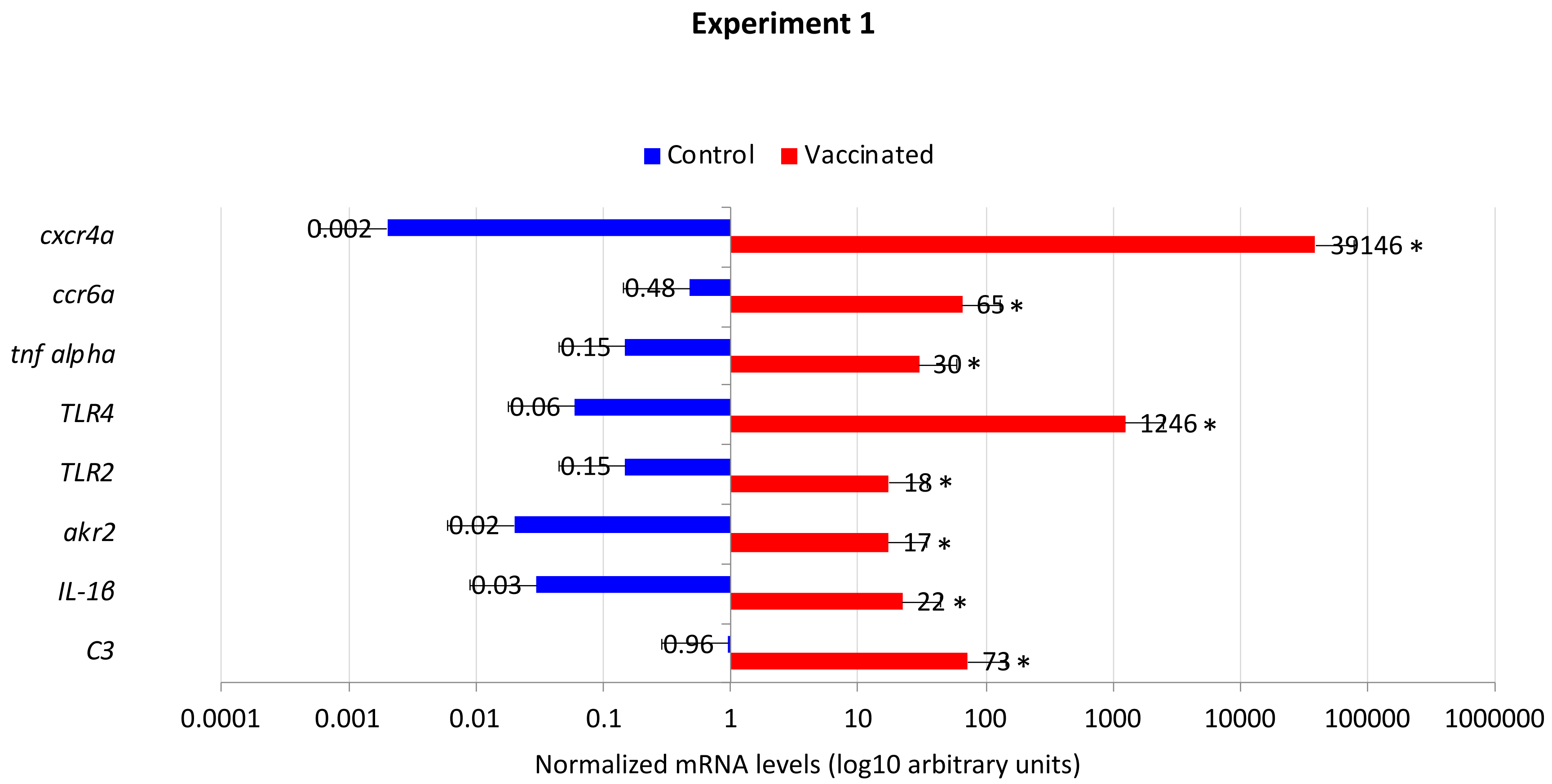

Figure S1A. Effect of zebrafish vaccination with α-Gal and mycobacterial infection on the expression of immune response genes. The expression of selected immune response genes was characterized by qRT-PCR in zebrafish in response to vaccination with BSA-α-Gal (α-Gal) infection with *M. marinum*. The mRNA levels were normalized against *D. rerio gapdh*, represented as average + S.D., and compared between groups by Student's t-test with unequal variance (*p < 0.05). (A) Experiment 1 with fish vaccinated with adjuvated BSA-α-Gal (N = 6) or BSA alone as control (N = 8) and challenged with IP *M. marinum*.

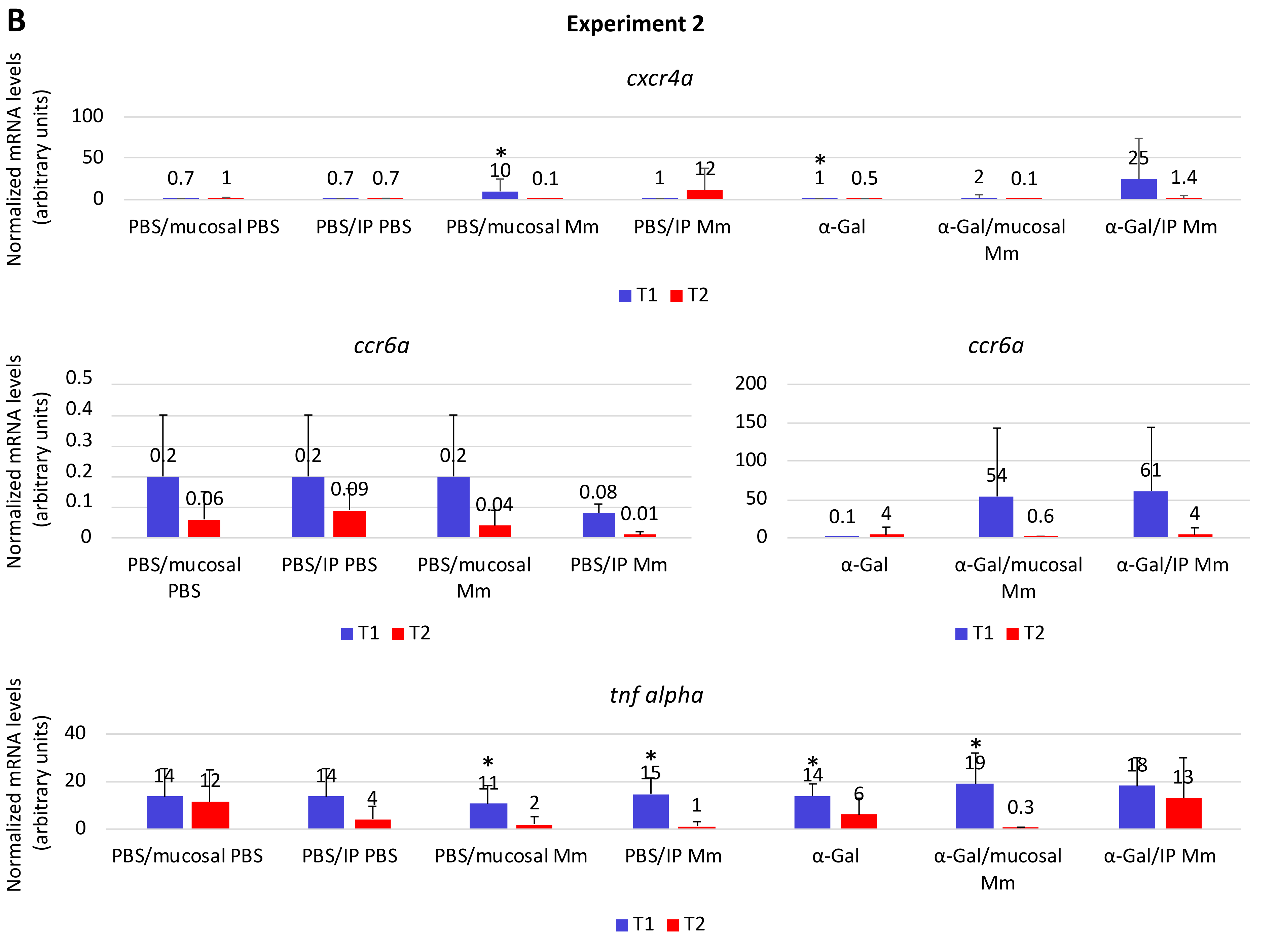
B
Experiment 2
cxcr4a
Normalized mRNA levels (arbitrary units)
100
50
0
0.7
1
0.7
0.7
*
10
0.1
1
12
*
1
0.5
2
0.1
25
1.4
PBS/mucosal PBS
PBS/IP PBS
PBS/mucosal Mm
PBS/IP Mm
α-Gal
α-Gal/mucosal Mm
α-Gal/IP Mm
T1 T2
ccr6a
0.5
0.4
0.3
0.2
0.1
0
0.2
0.06
0.2
0.09
0.2
0.04
0.08
0.01
PBS/mucosal PBS
PBS/IP PBS
PBS/mucosal Mm
PBS/IP Mm
T1 T2
ccr6a
200
150
100
50
0
0.1
4
54
0.6
61
4
α-Gal
α-Gal/mucosal Mm
α-Gal/IP Mm
T1 T2
tnf alpha
40
20
0
14
12
14
4
*
11
2
*
15
1
*
14
6
*
19
0.3
18
13
PBS/mucosal PBS
PBS/IP PBS
PBS/mucosal Mm
PBS/IP Mm
α-Gal
α-Gal/mucosal Mm
α-Gal/IP Mm
T1 T2

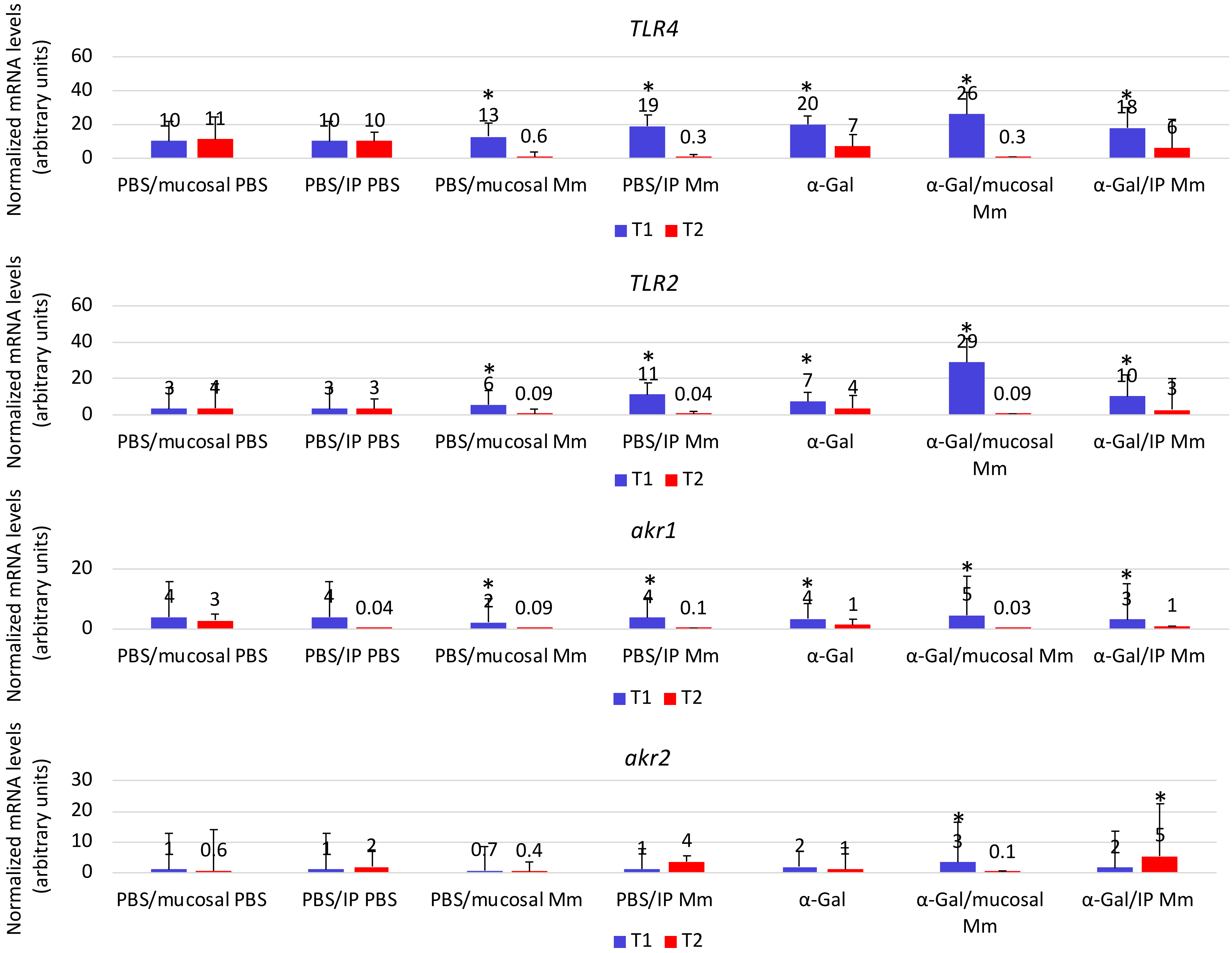

TLR4
Normalized mRNA levels (arbitrary units)
60
40
20
0
10
11
10
10
*
13
0.6
*
19
0.3
*
20
7
*
26
0.3
*
18
6
PBS/mucosal PBS
PBS/IP PBS
PBS/mucosal Mm
PBS/IP Mm
α-Gal
α-Gal/mucosal Mm
α-Gal/IP Mm
T1
T2
TLR2
Normalized mRNA levels (arbitrary units)
60
40
20
0
3
4
3
3
*
6
0.09
*
11
0.04
*
7
4
*
29
0.09
*
10
3
PBS/mucosal PBS
PBS/IP PBS
PBS/mucosal Mm
PBS/IP Mm
α-Gal
α-Gal/mucosal Mm
α-Gal/IP Mm
T1
T2
akr1
Normalized mRNA levels (arbitrary units)
20
0
4
3
4
0.04
*
2
0.09
*
4
0.1
*
4
1
*
5
0.03
*
3
1
PBS/mucosal PBS
PBS/IP PBS
PBS/mucosal Mm
PBS/IP Mm
α-Gal
α-Gal/mucosal Mm
α-Gal/IP Mm
T1
T2
akr2
Normalized mRNA levels (arbitrary units)
30
20
10
0
1
0.6
1
2
0.7
0.4
1
4
2
1
*
3
0.1
2
*
5
PBS/mucosal PBS
PBS/IP PBS
PBS/mucosal Mm
PBS/IP Mm
α-Gal
α-Gal/mucosal Mm
α-Gal/IP Mm
T1
T2

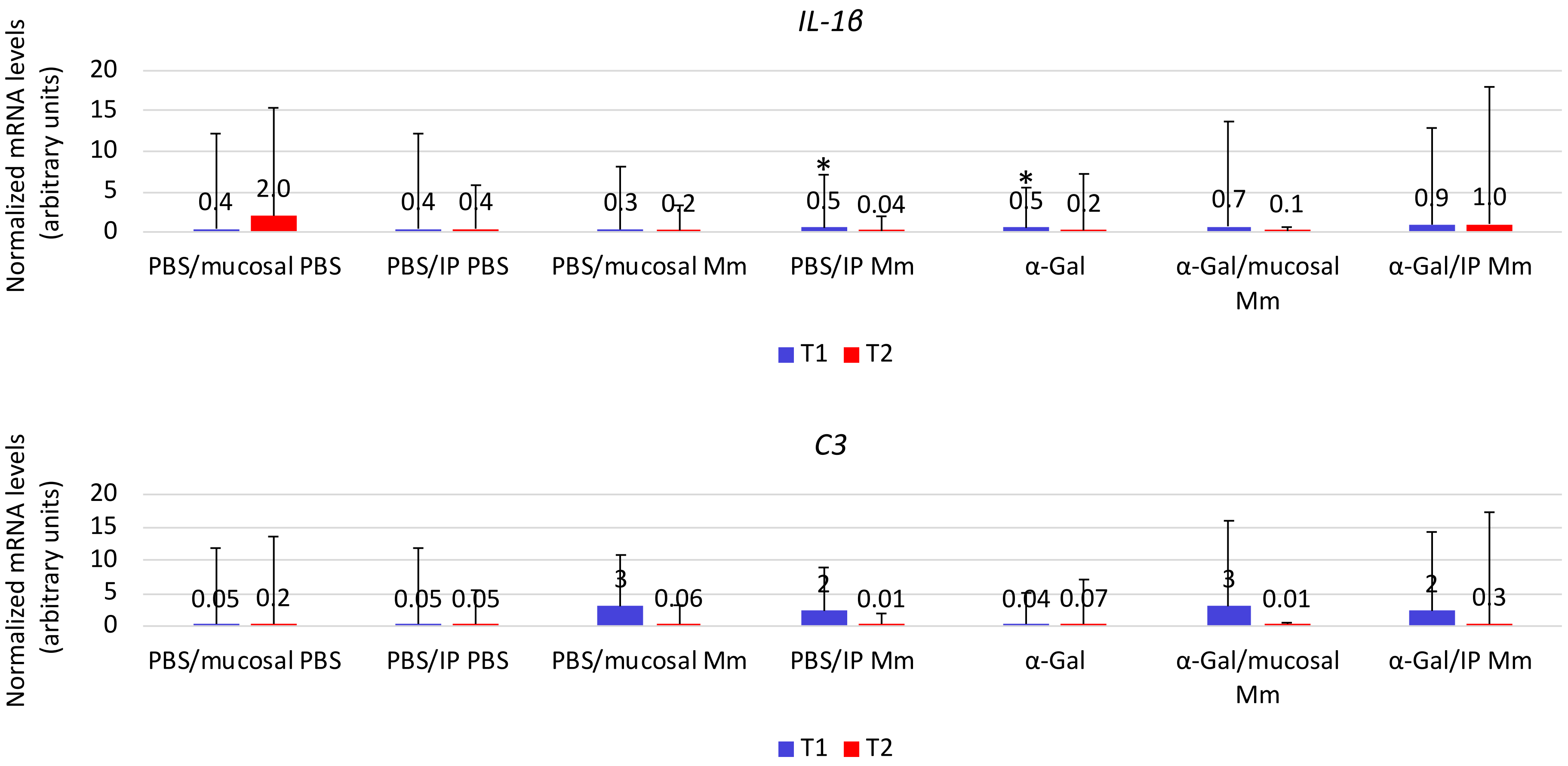

Figure S1B. Effect of zebrafish vaccination with α-Gal and mycobacterial infection on the expression of immune response genes. The expression of selected immune response genes was characterized by qRT-PCR in zebrafish in response to vaccination with BSA-α-Gal (α-Gal) infection with *M. marinum*. The mRNA levels were normalized against *D. rerio gapdh*, represented as average + S.D., and compared between groups by Student's t-test with unequal variance (*p < 0.05). (B) Experiment 2 with fish vaccinated with BSA-α-Gal (α-Gal) and PBS-treated controls uninfected and infected with mucosal or IP Mm at T2 (N = 5 for fish PBS vaccinated and IP PBS, N = 6 for fish PBS vaccinated and mucosal PBS, N = 8 for fich BSA-α-Gal vaccinated and untreated, N = 6 for fish vaccinated and IP Mm, N = 8 for fish vaccinated and mucosal Mm, N = 7 for controls and IP Mm, N = 10 for controls and mucosal Mm).

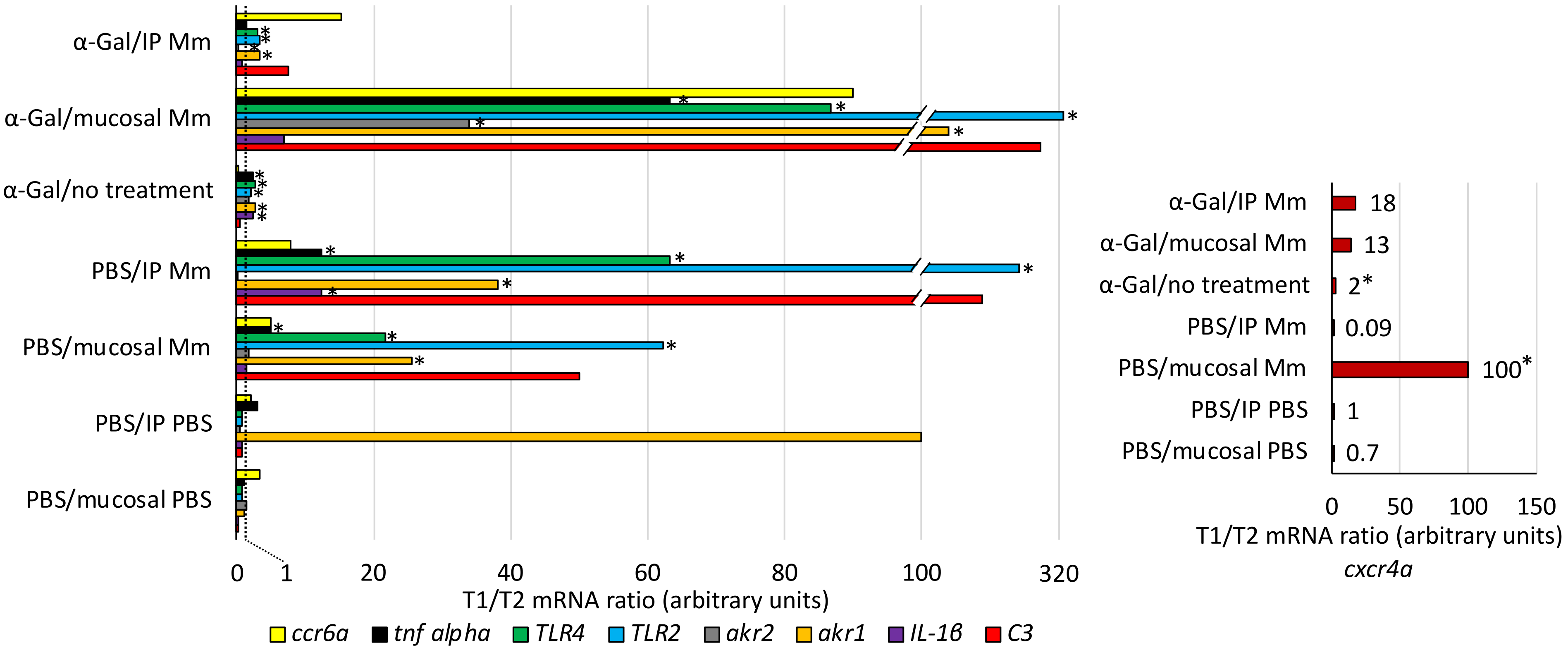

Figure S2. Effect of different treatments/vaccination and mycobacterial infection on the T1 to T2 mRNA ratio of immune response genes . The expression of selected immune response genes was characterized by qRT-PCR in Experiment 2 in zebrafish in response to PBS treatment or vaccination with α-Gal and IP/mucosal infection with *M. marinum* (Mm). The mRNA levels were normalized against *D. rerio gapdh* and normalized Ct values were compared between groups by Student's t-test with unequal variance (*p < 0.05; Figure S1B) and then represented as the T1/T2 ratio between normalized Ct values (T1, N = 7 for PBS vaccinated uninfected fish, N = 5 for α-Gal vaccinated uninfected fish, N = 13 for α-Gal vaccinated infected fish, N = 12 for PBS vaccinated infected fish; T2, N = 5 for fish PBS vaccinated and IP PBS, N = 6 for fish PBS vaccinated and mucosal PBS, N = 8 for fish α-Gal vaccinated and untreated, N = 6 for fish α-Gal vaccinated and IP Mm, N = 8 for fish α-Gal vaccinated and mucosal Mm, N = 7 for controls and IP Mm, N = 10 for controls and mucosal Mm).

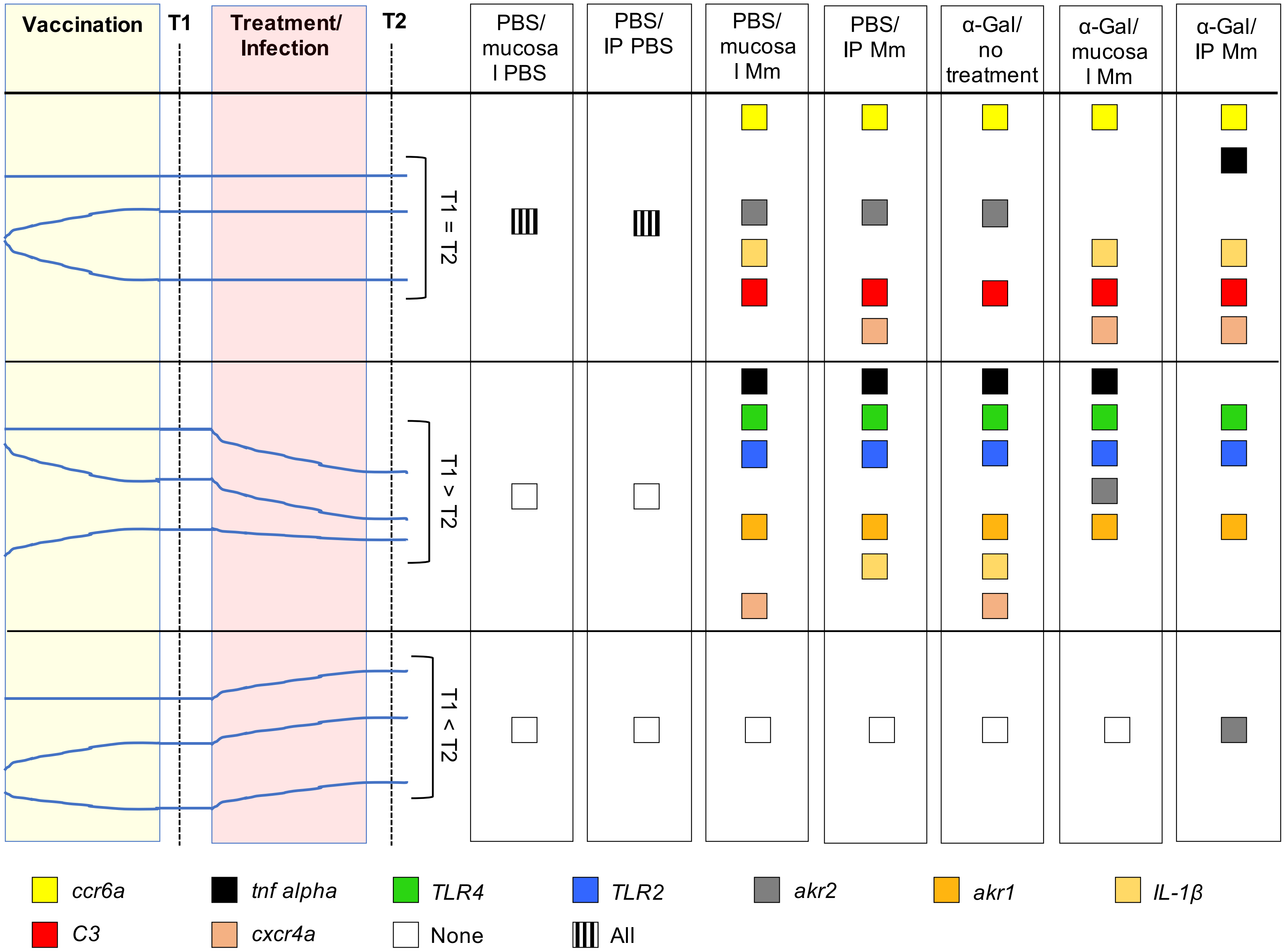

Figure S3. mRNA profile of immune response genes in response to different treatments/vaccination and mycobacterial infection. Based on the expression of selected immune response genes in Experiment 2 in zebrafish in response to PBS treatment or vaccination with α-Gal and IP/mucosal infection with *M. marinum* (Mm) (Figures S1B and S2), an mRNA profile was proposed based on all possible profiles resulting in values with T1 = T2, T1 > T2 and T1 < T2.

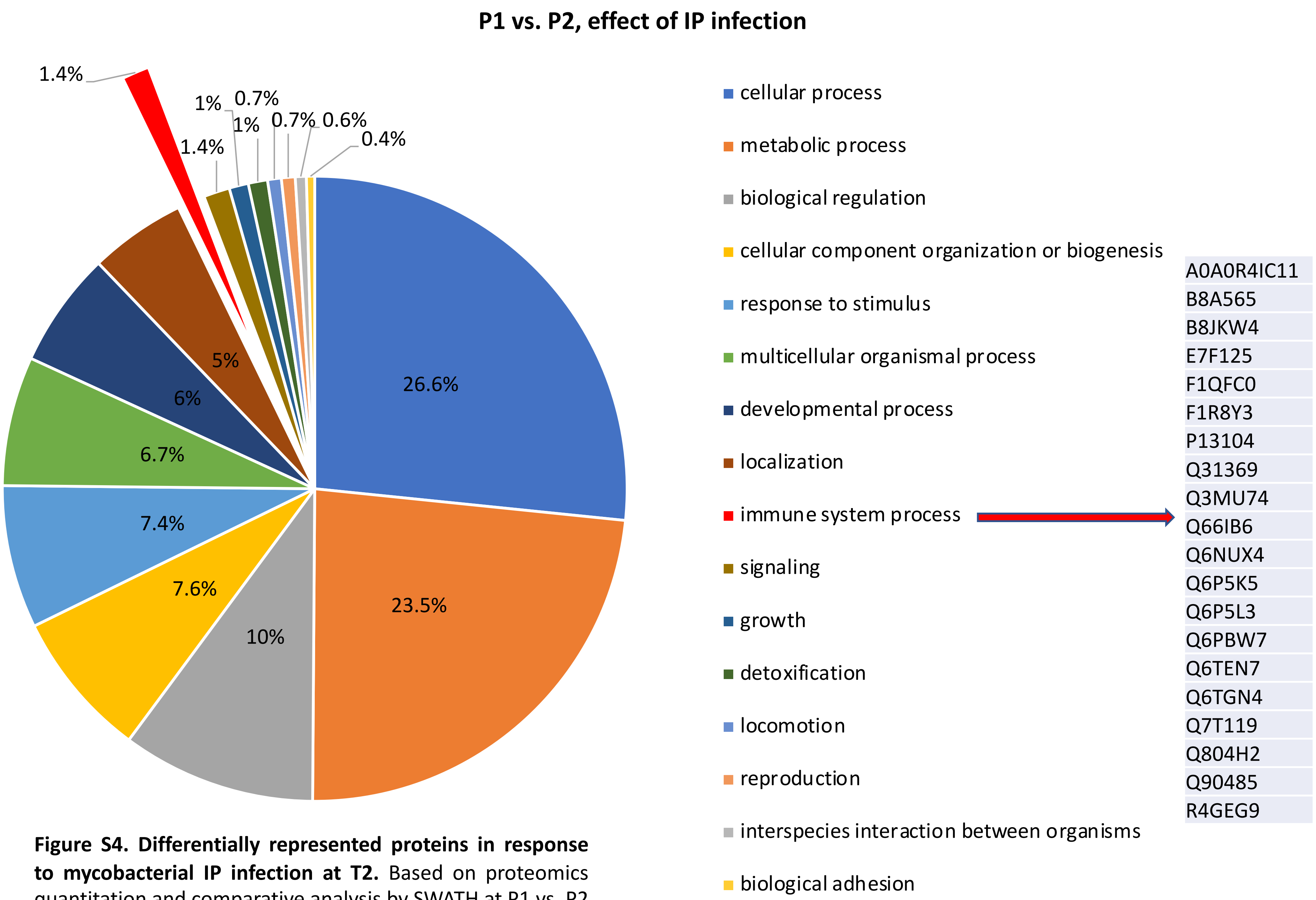

Figure S4. Differentially represented proteins in response to mycobacterial IP infection at T2. Based on proteomics quantitation and comparative analysis by SWATH at P1 vs. P2 (Figure 1B). Uniprot entries for immune system process annotated proteins are shown.

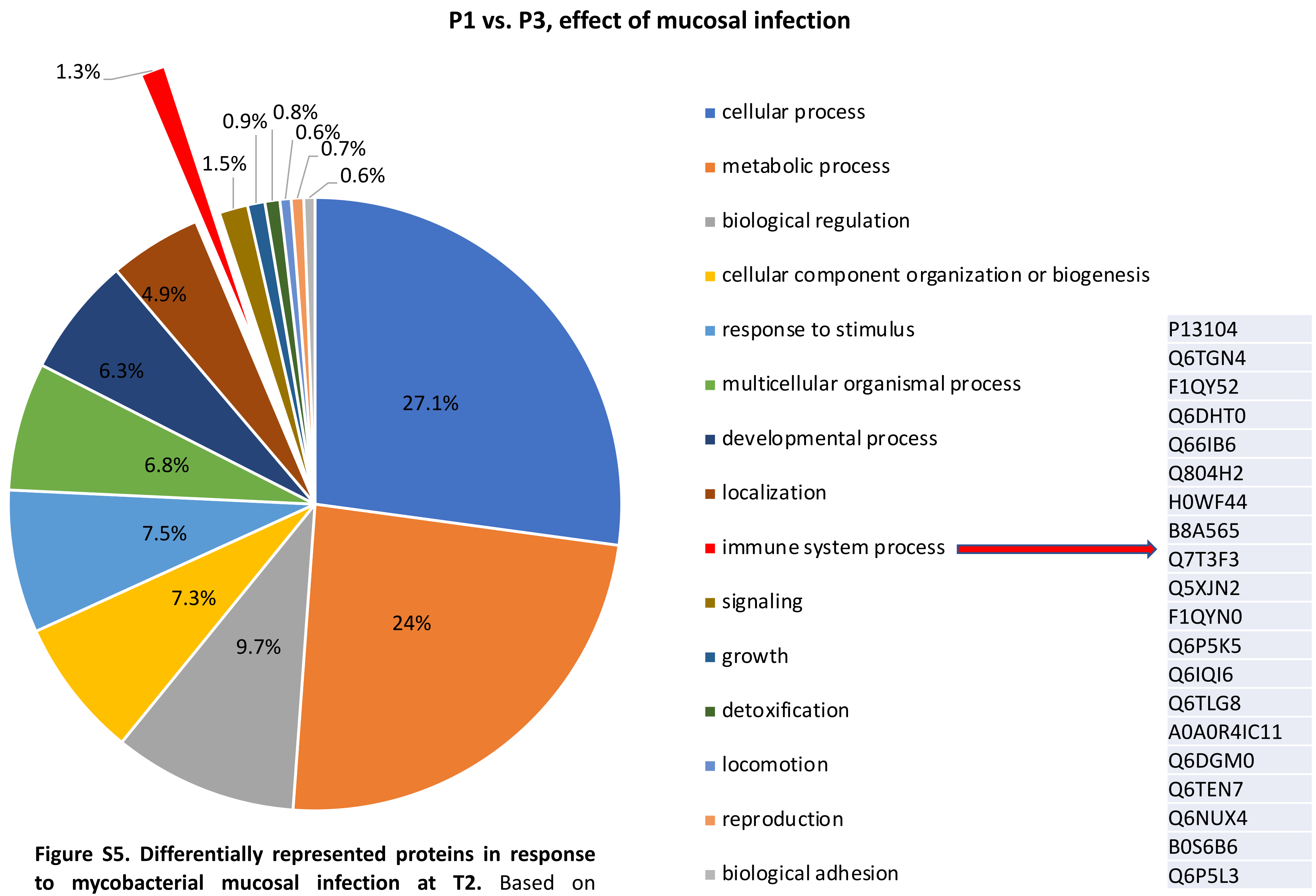

Figure S5. Differentially represented proteins in response to mycobacterial mucosal infection at T2. Based on proteomics quantitation and comparative analysis by SWATH at P1 vs. P3 (Figure 1B). Uniprot entries for immune system process annotated proteins are shown.

P1 vs. P4, effect of vaccination

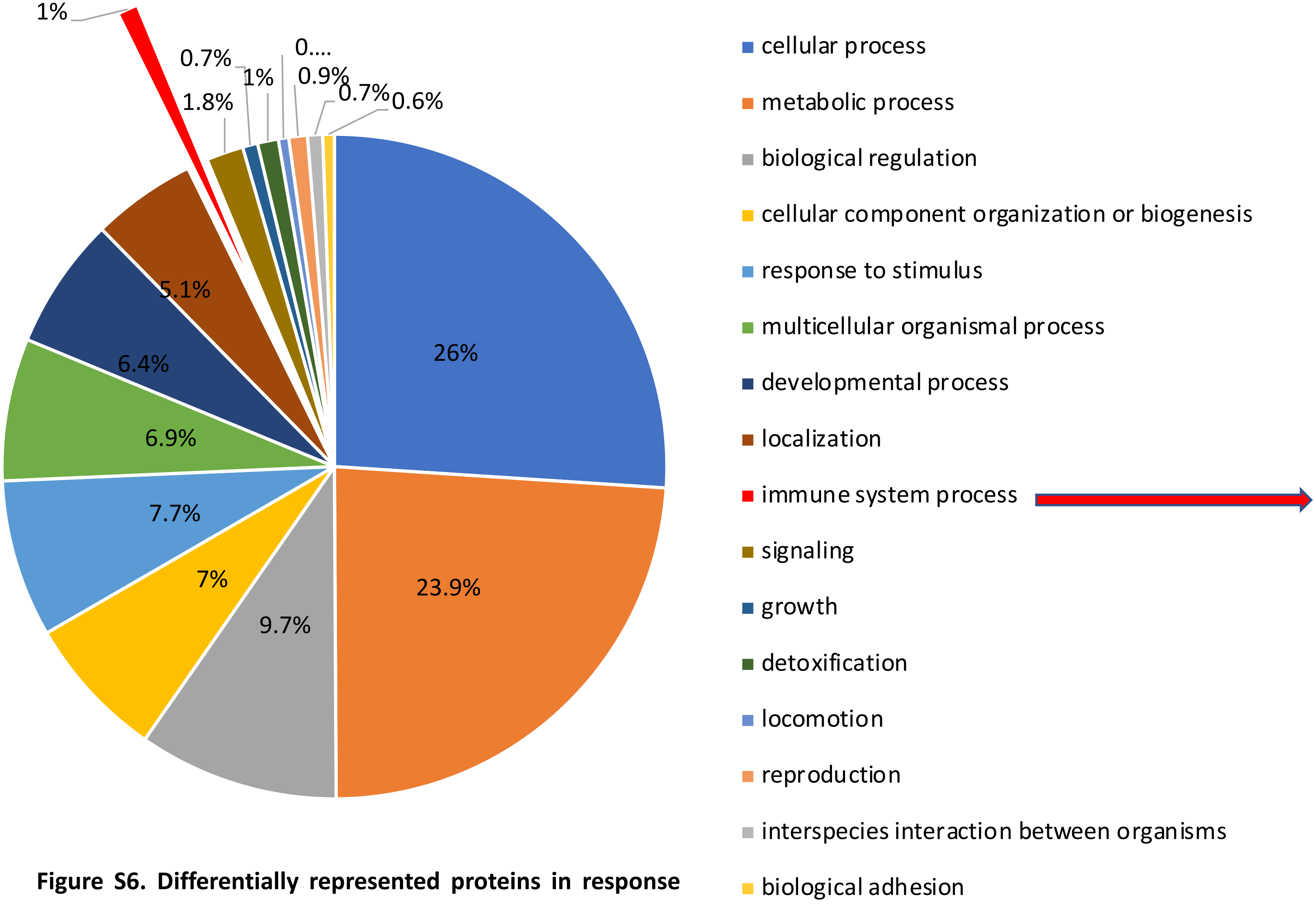

B8JKW4
F1QFC0
F1QW66
F1R264
P13104
Q0VFW0
Q3MU74
Q5BJJ3
Q66IB6
Q6DGM0
Q6DHT0
Q6IQN5
Q6NUX4
Q6P5K5
Q6TLG8
Q7T3F3
Q804H2
Q90485

Figure S6. Differentially represented proteins in response to vaccination with α-Gal at T1. Based on proteomics quantitation and comparative analysis by SWATH at P1 vs. P4 (Figure 1B). Uniprot entries for immune system process annotated proteins are shown.
